# Compression of Ribavirin to 35 GPa

**DOI:** 10.1021/acs.cgd.6c00376

**Published:** 2026-05-14

**Authors:** Bhaskar Tiwari, Simon Parsons, Nico Giordano

**Affiliations:** a Centre for Science at Extreme Conditions, School of Chemistry, 3124 The University of Edinburgh, Edinburgh EH9 3FJ, U.K.; b 28332 Deutsches Elektronen-Synchrotron DESY, Notkestr. 85, Hamburg 22607, Germany

## Abstract

The antiviral pharmaceutical compound ribavirin has two known crystalline polymorphs, **V1** and **V2**, under ambient conditions. The **V2** form is thermodynamically favored and more readily obtained. The higher-density **V1** form was obtained by high-pressure, high-temperature recrystallization in a diamond anvil cell. Recovered single crystals were compressed to 35.1 GPa and characterized by X-ray diffraction. The orthorhombic phase (*P*2_1_2_1_2_1_) is retained throughout compression, with changes in structural response near 10.9 and 26.6 GPa reflected by variations in *c*-axis compressibility and ribofuranosyl ring conformation. Compression proceeds from interlayer void collapse to progressive layer flattening and ultimately to intramolecular conformational adjustment once residual free volume is exhausted. The principal hydrogen-bond topology is preserved to the highest pressures investigated.

## Graphical abstract

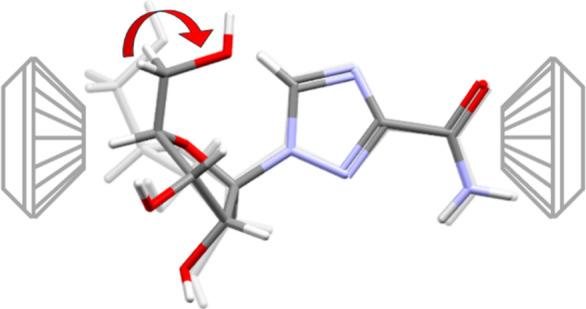

## 1 Introduction

While high-pressure studies can extend well into the megabar range, single-crystal studies of small organic molecules have tended toward a practical upper pressure limit of ∼ 10 GPa.^1,2^ This empirical boundary reflects a combination of experimental constraints, including the weak scattering of X-rays by light elements (C, H, N, O) in small or disordered crystals;^3^ hydrostatic limits of common pressure-transmitting media (PTMs);^4^ the geometrical restrictions imposed by the diamond anvil cell (DAC) design,^5^ and the limited flux and divergence of conventional laboratory X-ray sources. Dedicated extreme-conditions beamlines at synchrotron light sources mitigate many of these limitations by providing high-intensity, tunable hard X-rays and tightly focused microbeams, improving data completeness and reducing parasitic scattering from the DAC assembly.^6^

Despite these advances, single-crystal X-ray diffraction (SC-XRD) studies beyond 10 GPa remain rare for organic molecular systems. A search of the Cambridge Structural Database (CSD, v6.0, August 2025) restricted to entries in the high-pressure subset containing three-dimensional atomic coordinates determined by SC-XRD yields 2114 entries. Of these, 1959 (93%) were collected below 10 GPa, and only 57 (2.7%) extend beyond 20 GPa (Table S1 and Figure S1), highlighting the relative scarcity of single-crystal measurements in this regime. Structures refined above 20 GPa are typically determined using synchrotron radiation, reflecting the reliance of such studies on extreme-conditions beamlines. The regime above 10 GPa provides an opportunity to examine how molecular crystals accommodate compression when lower-pressure structural responses may no longer provide a complete description. As pressure increases, the relative importance of changes in void space, intermolecular contacts, hydrogen-bond geometry, molecular conformation, packing arrangement and phase stability may evolve. Single-crystal structural data under these conditions may therefore provide direct insight into phase evolution, limits to intermolecular interactions, and the ability of molecular crystals to preserve long-range order under extreme conditions.

Among the relatively few complex molecules refined above 20 GPa, most are π-conjugated or aromatic systems that adopt densely packed herringbone or layered arrangements under compression. These include pyrene (35.5 GPa),^7^ benzo­[*a*]­pyrene (27.9 GPa),^8^ anthracene (42 GPa) and naphthalene (51 GPa),^9^ as well as fluoroaromatic cocrystals—naphthalene·octafluoronaphthalene and anthracene·octafluoronaphthalene—which undergo polymerization near 20 and 25 GPa.^10^ Zhou et al. (refs 7–9 ) infer that the unexpected conformational flexibility of pyrene enables the pyrene-V phase to remain stable up to 35 GPa despite its nominally rigid aromatic framework, while the stabilities of naphthalene and anthracene polymorphs are attributed to the flexibility of the H···H and CH···π mediated herringbone motifs, which accommodate anisotropic compression and preserve single crystallinity to at least 50 GPa. In a more recent study, bibenzyl was refined to 37 GPa and exhibits a pressure-induced phase transition near 13 GPa, where molecular distortion is facilitated by torsional freedom of the saturated linker and accompanied by strengthening π···π interactions.^11^ While aromatic systems exhibit remarkable stability under compression, hydrogen-bonded frameworks are comparatively less explored. Studies of hydrogen-bonded molecular systems above 20 GPa remain rare, with examples in the CSD high-pressure subset (Table S1) limited to 4-hydroxybenzonitrile·helium and l-threonine.^12,13^ Additional reports of hydrogen-bonded systems above 20 GPa exist in the literature but are not represented in the CSD, for example melamine (1,3,5-triazine-2,4,6-triamine).^14^

The present work explores the effect of pressure to 35 GPa on the **V1** form of ribavirin, a pharmaceutically relevant ribofuranosyl-triazole derivative whose structure features a conformationally flexible ribose component and an extended hydrogen-bonded network. Understanding how functionalized active pharmaceutical ingredients (APIs) respond to compression is important for fundamental studies of polymorphism and for practical considerations in drug formulation and processing, where polymorphic form can influence mechanical performance and stability.^15^ High-pressure investigations of APIs have provided valuable insights into phase behavior and recrystallization, as reviewed elsewhere.^16^ Modest pressures can induce recrystallization from solution, for example, piracetam crystallizes as its form IV polymorph at only 0.07 GPa in methanol or around 0.4 GPa in water,^17^ while typical tablet-compaction processes subject APIs to pressures of approximately 0.1–0.3 GPa,^18^ highlighting the practical importance of high-pressure polymorph screening in pharmaceutical manufacturing. Beyond this processing-relevant pressure range, multi-GPa compression can be used to probe the wider solid-form landscape, test the stability limits of known polymorphs, and access high-pressure forms that may not be obtainable under ambient conditions. Most high-pressure crystallization studies of pharmaceuticals, summarized by Guerain,^16^ occur below 1 GPa, while reports of direct compression studies above 10 GPa are limited to powder X-ray diffraction and spectroscopic methods. Representative examples include indomethacin (13.7 GPa)^19^ and paracetamol (23 GPa).^20^ More recently, lamivudine has been examined by *in situ* Raman and FT-IR spectroscopy to 10.6 GPa,^21^ revealing reversible conformational changes, and the **V2** polymorph of ribavirin has been characterized by SC-XRD to ∼ 10 GPa,^22^ also showing reversible conformational changes.

Ribavirin has two known ambient-pressure crystalline polymorphs, **V1** and **V2**, both crystallizing in the orthorhombic space group *P*2_1_2_1_2_1_.^23^ The **V2** form is thermodynamically stable under ambient conditions,^24^ whereas the denser **V1** polymorph has proven difficult to isolate by conventional slow evaporation of solutions at room temperature. Literature reports, summarized in ref.^25^ and references therein, indicate that **V1** can only be accessed under nonambient conditions, including antisolvent recrystallization at 40 °C, solvent-mediated transformation with seeding (60–65 °C), recrystallization from an amorphous phase at 100 °C, or extended ball milling, illustrating the difficulty of obtaining this metastable phase under conventional crystallization conditions.

In this study, ribavirin **V1** was obtained via high-pressure, high-temperature (HP–HT) recrystallization in a DAC from either a melt or a solvent slurry. Recovered single crystals were subsequently compressed in a quasi-hydrostatic neon PTM to 35.1 GPa to examine structural evolution under extreme pressure. High-pressure crystal structure data are often interpreted in terms of the flexibility of intermolecular interactions, as in recent work on naphthalene and anthracene.^9^ However, as demonstrated in our previous study of the **V2** form of ribavirin,^22^ as well as in amino acids,^13,26,27^ and the highly polymorphic system ROY,^28^ intramolecular torsional flexibility can also play an important role in determining the phase behavior of molecular materials. This is also the case for ribavirin **V1**, where conformational flexibility enables continued compression without loss of crystallinity.

## 2 Experimental Section

### 2.1 Diamond Anvil Cell Preparation

High-pressure recrystallization experiments were performed in Merrill–Bassett type DACs, while compression experiments were carried out in short-symmetric DACs. Cells were equipped with Type Ia Boehler-Almax diamond anvils with culet diameters of 500 and 400 μm, respectively. Rhenium gaskets were preindented to 65–85 μm, and circular sample chambers were drilled by spark erosion with initial diameters of approximately half the corresponding culet diameter. Pressures were determined using the ruby fluorescence method with the IPPS-Ruby2020 calibration,^29^ giving estimated uncertainties of ± 2.5%.

### 2.2 Preparation and Compression of Ribavirin V1

Ribavirin (≥98%, Sigma-Aldrich) was used as received and identified as the **V2** polymorph by SC-XRD. The **V1** polymorph was obtained using three approaches: (i) HP–HT recrystallization from the melt, (ii) recrystallization from a high-pressure solvent slurry, and (iii) seeding under ambient conditions. A fourth experiment (iv) examined the compression behavior of recovered crystals.

#### 2.2.1 Recrystallization from the Melt

Ribavirin was ground into a fine powder using a mortar and pestle and packed into the DAC sample chamber along with ruby spheres for pressure determination. The cell was closed to approximately 0.3 GPa without a PTM. The DAC was gradually heated to ∼ 185 °C using a heat gun until partial melting was observed, then maintained at this temperature until nearly all solid material had melted. Temperatures were monitored periodically using a thermocouple attached to the diamond table. The cell was then left to stand and allowed to cool to room temperature.

#### 2.2.2 Recrystallization from a Ribavirin-Zidovudine Slurry

In an experiment initially aimed at producing a ribavirin-zidovudine cocrystal, an equimolar mixture of ribavirin (0.03962 g, 0.162 mmol) and zidovudine (0.04329 g, 0.162 mmol) was ground with a mortar and pestle to obtain a homogeneous powder. The powder was slurried with ethanol–acetone (1:1 v/v; 0.20 mL) and loaded into a DAC along with ruby spheres for pressure determination. The DAC was closed at 0.8 GPa and heated on a hot plate until the contents became optically transparent (apparent dissolution), after which it was allowed to cool to room temperature.

#### 2.2.3 Crystallization by Seeding under Ambient Conditions

A crystal of ribavirin **V1**, obtained from the melt experiment (i), was placed on a microscope well slide. A single drop of a concentrated solution of ribavirin in ethanol was added to cover the seed. Crystallization proceeded as the solvent evaporated under ambient conditions.

#### 2.2.4 Compression Study

Two single crystals of ribavirin **V1**, recovered from the ribavirin–zidovudine recrystallization experiment (ii), were used in independent compression studies. Each crystal was loaded into a DAC together with ruby spheres for pressure determination. In the first experimental run, the sample was compressed stepwise to 12 GPa with diffraction measurements performed at selected pressures before termination due to gasket failure. In the second run, the cell was compressed stepwise to 9 GPa and diffraction measurements were then performed from 9 to 35 GPa at selected pressure intervals. Two independently measured structures at 9.1 GPa showed close agreement in unit-cell parameters, supporting reproducibility across the overlapping pressure range. Neon, loaded using a Sanchez Technologies gas-loading system, served as the pressure-transmitting medium.

### 2.3 High Pressure Single Crystal X-ray Diffraction

All SC-XRD measurements were performed at the P02.2 Extreme Conditions Beamline of PETRA III (DESY, Hamburg, Germany) using synchrotron radiation (λ = 0.2920 and 0.2906 Å; beam size ≈ 8 × 3 μm^2^ full width at half-maximum) and a PerkinElmer XRD1621 detector.^30^ Data were collected using a single-axis (ω) rotation stage. For samples measured in the DAC, ω scans spanned – 35 ° to +35 ° in 0.5 ° increments with 1 s exposure per frame, these limits being defined by the DAC geometry. Recovered crystals were either retained in the gasket or transferred to a diamond plate holder with a larger opening angle than the DAC. The as-supplied material was mounted in the same manner. All measurements used a ± 35 ° ω range and identical exposure times for consistency.

### 2.4 Structure Solution and Refinement

The diffraction data were integrated and reduced using CrysAlisPro.^31^ Absorption corrections were applied using SCALE3 ABSPACK to account for systematic errors, including absorption effects and gasket shading. Structure solutions were carried out using the dual space method (SHELXT),^32^ and refinements were performed against |*F*|^2^ using SHELXL,^33^ within the Olex2 graphical interface.^34^ Non-hydrogen atoms (C, N, and O) were refined with anisotropic displacement parameters, while enhanced rigid-bond restraints (RIGU) were applied at high pressure to stabilize the refinement of these parameters.^35^ Hydrogen atoms were placed on carbon and nitrogen in idealized geometries and refined using a riding model; those on hydroxyl groups in the ambient-pressure structure were initially refined freely, then allowed to ride on their parent atoms. The same model was applied to all high-pressure refinements. Data completeness across the compression series ranged from 80.2 to 61.0%. The highest *R*-factor (*R*_1_) and weighted *R*-factor (*w*R_2_) were 7.6% and 23.8% respectively, corresponding to the structure measured at 35.1 GPa. Selected crystallographic and refinement statistics are summarized in Table 1, with a complete set provided in Table S2.

**Table 1:** Selected Crystallographic and Refinement Data for Ribavirin **V1** at Ambient and High Pressure^a^

pressure (GPa)	ambient	5.0	10.2	15.1	20.3	25.7	30.1	35.1
crystal data								
*a, b, c* (Å)	7.5198(5), 8.8235(8), 14.8847(6)	7.1504(6), 8.1138(4), 14.4756(5)	6.9309(7), 7.8128(5), 14.3156(6)	6.7698(8), 7.5096(3), 14.3577(7)	6.5913(9), 7.2836(4), 14.4829(9)	6.391(2), 7.1077(7), 14.6735(18)	6.234(3) 6.9771(11) 14.803(3)	6.093(5), 6.9256(18), 14.811(4)
*V* (Å^3^)	987.61(11)	839.83(9)	775.19(10)	729.92(9)	695.3(1)	666.6(2)	643.8(4)	625.0(6)
*Z*	4	4	4	4	4	4	4	4
data collection								
no. of measured, independent and observed [*I* > 2σ(*I*)] reflections	2380, 1188, 1138	1825, 1243, 1146	1755, 1173, 1020	1856, 1076, 1059	1595, 1011, 977	1502, 952, 858	1500, 918,783	1254, 792, 590
*R*_int_	0.015	0.047	0.048	0.029	0.022	0.037	0.044	0.050
refinement								
*R*[*F* ^2^ > 2σ(*F* ^2^)], *w*R(*F* ^2^), *S*	0.027 0.071 1.11	0.053, 0.155, 1.13	0.060, 0.181, 1.10	0.036, 0.086, 1.12	0.032, 0.086, 0.96	0.047, 0.132, 1.09	0.054, 0.148, 1.05	0.076, 0.238, 1.15

a The crystal retained the space group *P*2_1_2_1_2_1_ (*Z*′ = 1) throughout the pressure series.

### 2.5 Other Programs Used

Structure visualization and geometric analyses were performed using Mercury,^36^ while evaluation of bond lengths, angles, and torsional and ring fragments against the CSD were carried out using Mogul.^37^ The database search was performed separately in ConQuest.^38^ Ring-puckering analysis was conducted in PLATON.^39–41^

Intermolecular interaction and crystal lattice energies were calculated using CLP-PIXEL,^42–45^ via MrPixel in the Mercury interface.^46^ Electron-density calculations were performed at the MP2 level of theory with the 6–31G** basis set using Gaussian09.^47^ Additional intermolecular interaction energies for contacts within the first molecular coordination sphere were evaluated by Symmetry-Adapted Perturbation Theory (SAPT) at the SAPT0 level.^48,49^ These calculations were performed within PSI4 ^50^ employing the aug-cc-VDZ basis set, with molecular dimers identified in CrystalLattE^51^ using a maximum two-body closest contact distance of 3 Å.

Void-space and molecular-volume analyses were performed using CellVol and MolVol,^52^ which employ a Monte Carlo algorithm based on atomic van der Waals radii to quantify the volumes of isolated molecules and the occupied and unoccupied regions within crystal structures. Equation-of-state (EoS) fitting was carried out using EoSFIT7-GUI.^53^ Third-order Birch–Murnaghan EoSs were used to model the variation of total and occupied (“network”) volumes with pressure, while a Vinet EoS was applied to the void volumes.

## 3 Results and Discussion

### 3.1 Preparation of V1

Formation of the **V1** polymorph of ribavirin was investigated using three approaches.

i. Melt recrystallization within the DAC yielded a polycrystalline mass of ribavirin **V1** that completely filled the sample chamber (Figure 1a). In situ synchrotron SC-XRD confirmed that all indexed grains corresponded to the **V1** polymorph. After decompression, the recovered material was re-examined and likewise identified as **V1**; however, the fine-grained crystals were unsuitable for subsequent high-pressure SC-XRD experiments. These were instead used as seeds in ambient-pressure recrystallization in an attempt to obtain larger, better-formed crystals.
ii. High-pressure recrystallization from a ribavirin–zidovudine slurry in a 1:1 ethanol–acetone mixture yielded discrete single crystals of ribavirin **V1** rather than the targeted cocrystal (Figure 1b). In situ SC-XRD mapping of ten optically crystalline regions identified only the **V1** phase, and inspection of the remainder of the sample chamber showed no evidence of crystalline zidovudine, which likely remained dissolved or precipitated in an amorphous form. After decompression, the recovered crystals were confirmed to retain the **V1** structure. For comparison, recrystallization from ethanol slurries of pure **V2** under comparable HP–HT conditions produced only the **V2** form, whereas acetone alone was unsuitable owing to the low solubility of ribavirin in this solvent.^54^
iii. Crystallization by seeding under ambient conditions produced two distinct crystal morphologies (Figure 1c): block-shaped crystals identified as **V1**, and needle-like crystals corresponding to **V2**, both verified by SC-XRD. The appearance of **V2** likely reflects rapid nucleation from concentrated ethanol solution during solvent evaporation. Overall, the **V1** crystals obtained by seeding were smaller and of lower quality than those produced by the mixed-solvent high-pressure slurry method (ii).

**Figure 1:**
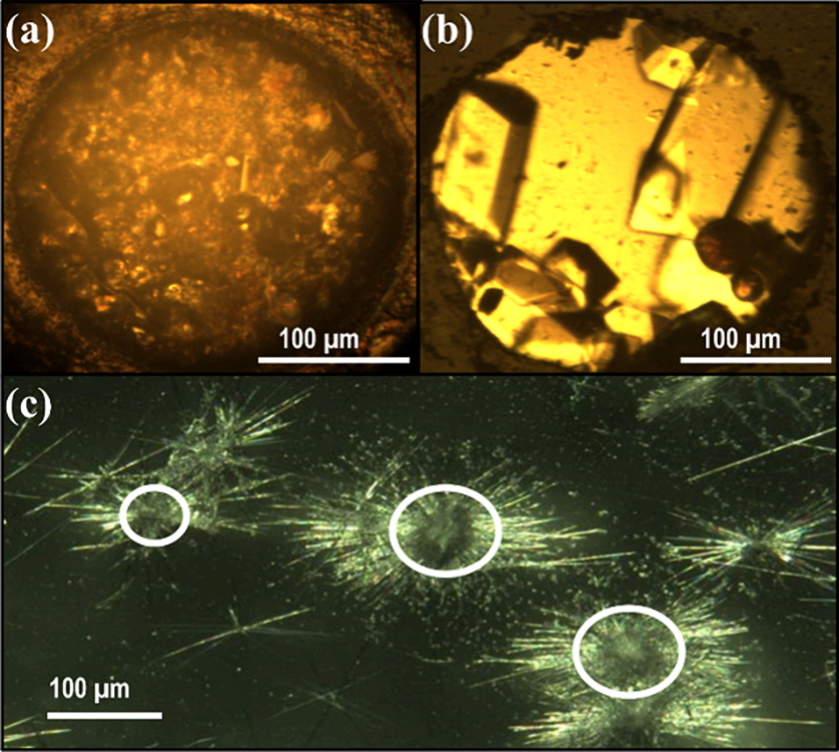
Recrystallization of the **V1** polymorph of ribavirin from the melt at ∼ 0.3 GPa (method (i). (b) Recrystallization of single crystals of ribavirin **V1** from a 1:1 ethanol–acetone mixture at ∼ 0.8 GPa (method (ii). (c) Crystallization of the **V1** (circled blocks) and **V2** polymorphs under ambient conditions (method (iii).

Single crystals obtained from the mixed-solvent high-pressure slurry recrystallization (ii) were of sufficient size and quality for subsequent compression studies. These were used for structural characterization at ambient pressure and for high-pressure SC-XRD.

### 3.2 The Structure of Ribavirin V1 at Ambient Pressure

Ribavirin is a guanosine analog, comprising a 1,2,4-triazole-3-carboxamide nucleobase linked to a β-D-ribofuranosyl unit via a C–N glycosidic bond (Figure 2a). The **V1** form crystallizes in the orthorhombic space group *P*2_1_2_1_2_1_, with one molecule in the asymmetric unit and four per unit cell. The atomic numbering scheme for non-hydrogen atoms follows the previously published structure in the CSD (Refcode VIRAZL),^23^ but the standard setting of the unit cell is used here (Figure 2b).

**Figure 2:**
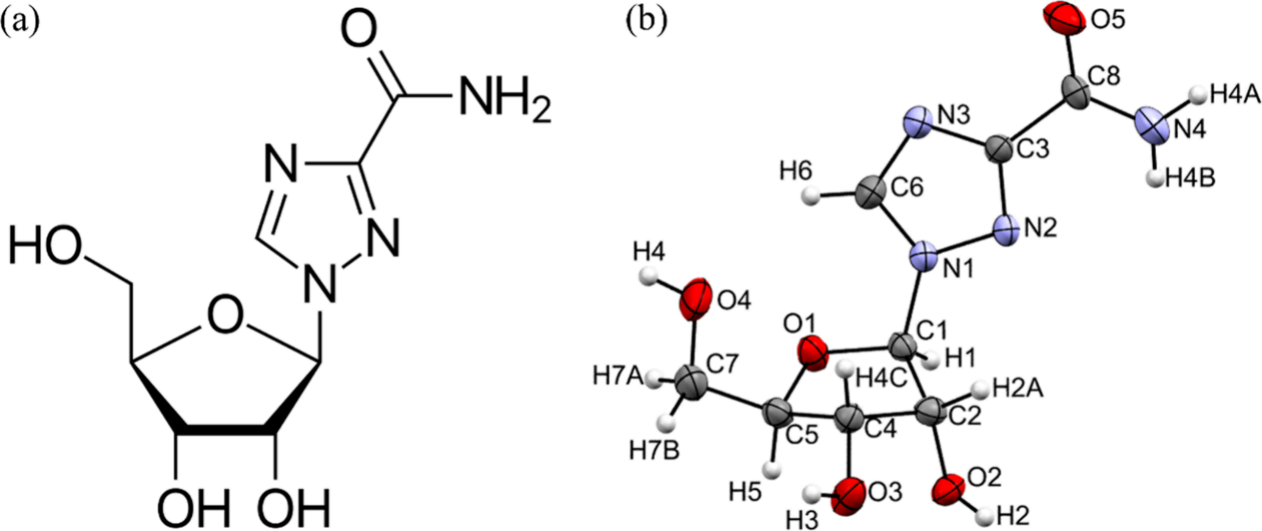
(a) Structural formula of ribavirin. (b) Molecular structure of the **V1** form at ambient pressure (determined in this study) showing the atomic numbering scheme. Ellipsoids are shown at the 50% probability level.

The ribofuranosyl ring conformation is described using the Altona–Sundaralingam pseudorotation formalism, expressed in terms of a pseudorotation phase angle (*P*) and puckering amplitude (τ_m_).^55^ Envelope (E) and twist (T) descriptors identify whether one atom, or two adjacent atoms, are displaced from the mean plane of the five-membered ring. The numerical labels follow the conventional furanose pseudorotation numbering, O0–C1–C2–C3–C4, which differs from the crystallographic numbering used here; this sequence corresponds to O1–C1–C2–C4–C5 in the present structure (Figure 2b). In this notation, superscripts denote displacement above the mean ring plane and subscripts denote displacement below it. At ambient pressure, the ribofuranosyl ring in **V1** adopts an E^3^ envelope conformation, with *P* = 11.1(2)°. In this conformation, C3 in the furanose pseudorotation numbering (C4 in this work) is displaced above the mean ring plane.

Crystal packing in **V1** is governed by directional hydrogen bonding supplemented by dispersion-dominated interactions, generating a corrugated layer motif within the first coordination sphere (Figure 3a). PIXEL analysis identifies 14 neighboring molecules in the first coordination sphere, represented by seven symmetry-unique molecule–molecule interactions (*A–G*) and their symmetry equivalents (*A′–G′*), with interaction energies listed in order of strongest to weakest cohesive energy at ambient pressure in Table 2. The total crystal lattice energy in **V1** is – 223.4 kJ mol^–1^, compared with – 227.8 kJ mol^–1^ for the more stable **V2** form.^22^ The strongest interactions (*A–C*), which are dominated by hydrogen bonding contacts, generate two-dimensional corrugated sheets in the *ac*-plane (Figure 3b), which stack along the *b*-axis through weaker, dispersion-dominated interactions (*D–G*).

**Figure 3:**
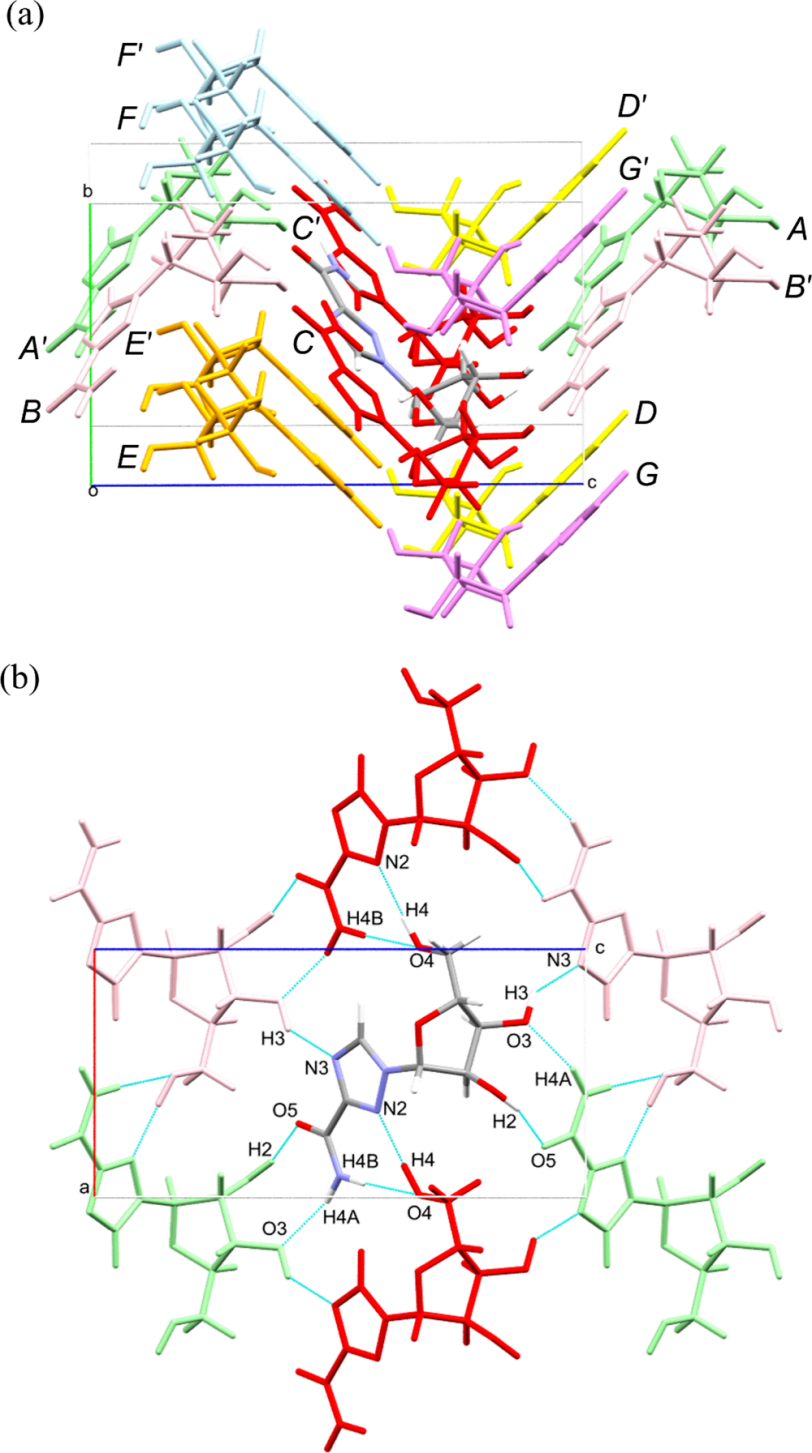
(a) First coordination sphere of ribavirin **V1** at ambient pressure, showing 14 molecules surrounding a central reference molecule (colored by element). The seven symmetry-unique interactions (*A*–*G*) and their symmetry equivalents (*A*′–*G*′) are individually labeled, with molecules color-coded by symmetry equivalence. These interactions are listed in Table 2. (b) Hydrogen bonding network formed by interactions *A*/*A*′–*C*/*C*′ in the ac-plane. The color scheme is as in (a): *A*/*A*′ (green), *B*/*B*′ (pink), and *C*/*C*′ (red).

**Table 2:** Intermolecular Interaction Energies within the First Coordination Sphere of Ribavirin **V1**, Calculated Using the PIXEL Method^a^

symmetry operation	label	centroid distance (Å)	Coulombic	polarization	dispersion	repulsion	total	contacts
–*x*+^3^/_2_, – *y*+1, *z*–^1^/_2_ – *x*+^3^/_2_, – *y*+1, *z*+^1^/_2_	*A/A′*	9.429	–80.0	–31.2	–21.3	81.4	–51.2	2 × N4H4A···O3 = 2.20 Å, ∠ = 151.9° 2 × O2H2···O5 = 1.90 Å, ∠ = 162.0°
–*x*+^1^/_2_, – *y*+1, *z*–^1^/_2_ – *x*+^1^/_2_, – *y*+1, *z*+^1^/_2_	*B/B′*	8.337	–53.7	–20.2	–15.6	41.9	–47.6	2 × O3H3···N3 = 2.17 Å, ∠ = 145.3°
*x*–1, *y*, *z x*+1, *y*, *z*	*C/C′*	7.520	–42.5	–24.4	–28.7	55.5	–40.1	2 × O4H4···N2 = 2.13 Å, ∠ = 161.4° 2 × N4H4B···O4 = 2.40 Å, ∠ = 174.4°
–*x*+1, *y*–^1^/_2_, – *z*+^3^/_2_ – *x*+1, *y*+^1^/_2_, – *z*+^3^/_2_	*D/D′*	5.326	–15.6	–6.9	–38.7	25.3	–35.9	dispersion dominant interaction
*x*–^1^/_2_, – *y*+^1^/_2_, – *z*+1 *x*+^1^/_2_, – *y*+^1^/_2_, – *z*+1	*E/E′*	6.262	–13.1	–5.5	–29.6	17.6	–30.6	dispersion dominant interaction
*x*+^1^/_2_, – *y*+^3^/_2_, – *z*+1 *x*–^1^/_2_, – *y*+^3^/_2_, – *z*+1	*F/F′*	9.443	–6.0	–5.1	–8.7	3.5	–16.3	nonspecific long-range interaction
–*x*, *y*–^1^/_2_, – *z*+^3^/_2_ – *x*, *y*+^1^/_2_, – *z*+^3^/_2_	*G/G′*	8.094	–3.5	–2.4	–10.9	4.0	–12.8	nonspecific long-range interaction

a Energies are reported in kJ mol^–1^. Centroid distance refers to the distance between the central reference molecule and each neighboring molecule. Hydrogen atom distances refer to ‘un-normalized’ positions obtained from the refinements against X-ray data.

Interaction *A* is the strongest (−51.2 kJ mol^–1^), dominated by electrostatic attraction from two hydrogen bonds between the carboxamide group and ribofuranosyl hydroxyl groups, forming an R_2_^2^(9) cyclic dimer. Interaction *B* (−47.6 kJ mol^–1^) comprises a single hydrogen bond between the ribofuranosyl O3H3 hydroxyl group and triazole N3. Interactions *A*/*A*′ and *B*/*B*′ generate corrugated ribbons along **c**, while interaction *C* (−40.1 kJ mol^–1^) extends these along **a** into two-dimensional sheets in the *ac*-plane. Interlayer cohesion is mediated by dispersion-dominated interactions (*D*–*G*). Among these, *D* (−35.9 kJ mol^–1^) and *E* (−30.6 kJ mol^–1^) provide the principal stacking cohesion, while *F* and *G* (−16.3 and – 12.8 kJ mol^–1^) represent weaker, longer-range contacts.

### 3.3 Pressure–Volume Behavior of Ribavirin V1

The ambient-pressure **V1** form of ribavirin persists to at least 35.1 GPa, retaining the orthorhombic *P*2_1_2_1_2_1_ space group throughout. Over this pressure range, the unit cell volume decreases by 36.7%, from 987.61(11) to 625.0(6) Å^3^ (Figure 4a). A third-order Birch–Murnaghan equation of state was fitted to pressure–volume data using a fixed zero-pressure volume of *V*_0_ = 987.61(11) Å^3^. The resulting bulk modulus is *K*_0_ = 19.0(3) GPa, with a first pressure derivative, *K′* = 6.5(1). **V1** is significantly less compressible than the **V2** polymorph (*K*_0_ = 13.7(7) GPa, *K′* = 9.2(1)),^22^ consistent with its higher ambient-pressure density.

**Figure 4:**
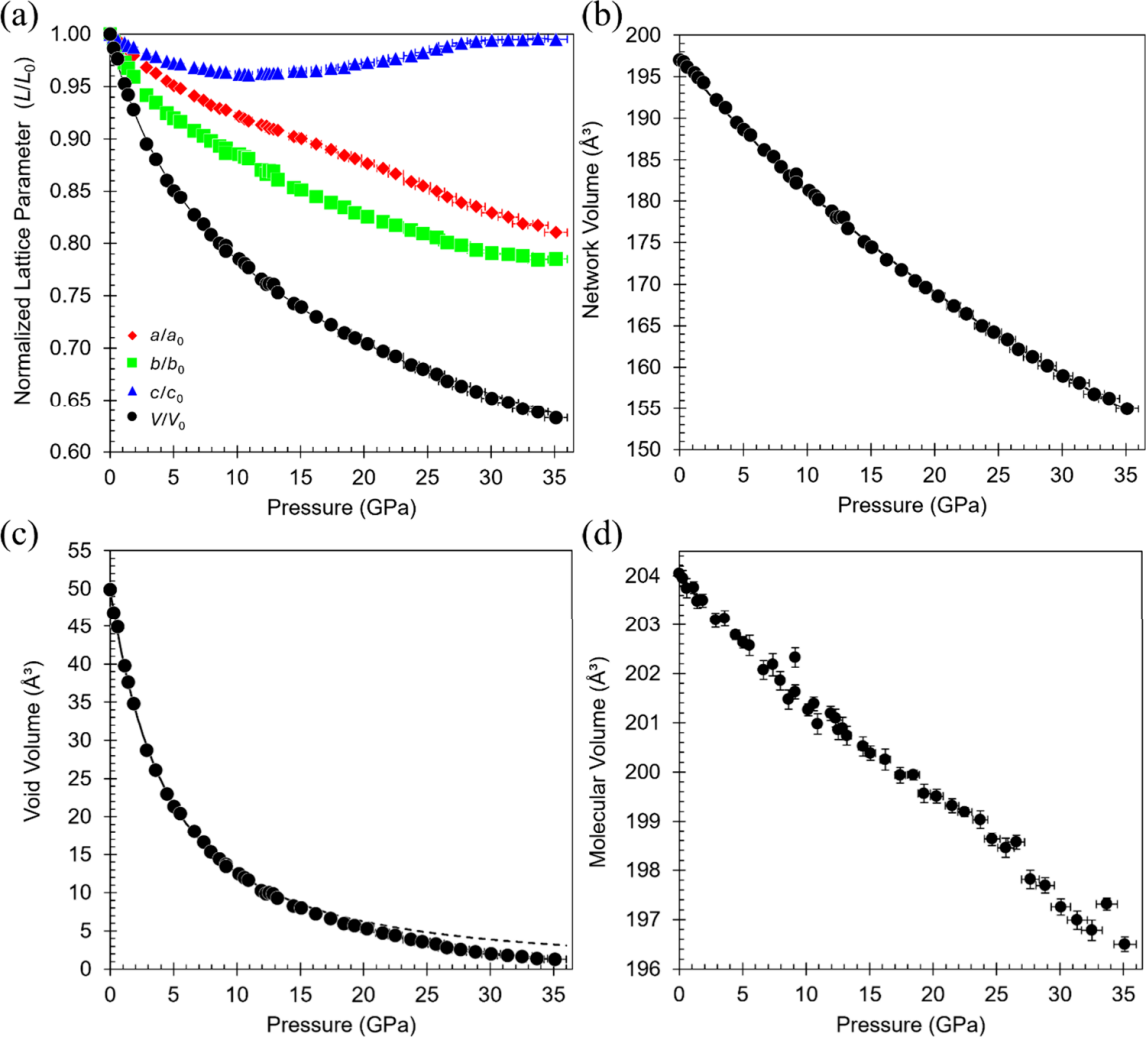
Compression behavior of ribavirin **V1**. (a) Normalized unit cell volume (*V*/*V*_0_) and lattice parameters (*L*/*L*_0_), (b) network volume, (c) void volume, and (d) molecular volume as a function of pressure. Solid lines indicate fitted EoS curves; the dashed line in (c) marks extrapolation beyond the fit range. Error bars are included for all data points; where not visible, they are smaller than or obscured by the symbols.

Owing to its orthorhombic symmetry, the principal directions of strain correspond to the crystallographic *a-*, *b-,* and *c*-axes of the unit cell (Figure 4a). Compression is markedly anisotropic across the entire pressure range. The largest strain occurs along the *b*-axis, corresponding to the layer-stacking direction, which contracts monotonically by 21.5% over the measured pressure range. The *a*-axis also contracts in an essentially monotonic fashion, shortening by 19.0%. The *c*-axis initially decreases by 3.9% to 10.9 GPa, then expands by 3.5% to 30.1 GPa, returning nearly to its original length before plateauing at higher pressures. Although no discontinuity is observed in the unit cell volume, the inflection in the *c*-axis indicates a change in compression mechanism near 11 GPa.

Further subtle features of the compression behavior become clearer upon partitioning the unit-cell volume per molecule (*V*_cell_/*Z*) into occupied and unoccupied space.^52^ The resulting trends are shown in Figure 4b,c. The network volume, representing the molecular framework, decreases smoothly with increasing pressure, reducing by 21.4% from 197.03(7) Å^3^ at ambient pressure to 154.96(2) Å^3^ at 35.1 GPa. This trend is well described by a third-order Birch–Murnaghan EoS (*K*_net_ = 106(1) GPa), indicating that the hydrogen-bonded framework remains structurally intact throughout compression. The void volume collapses by 97.4% over the same range and is best described by a third-order Vinet EoS only up to 10.2 GPa (*K*_void_ = 4.6(1) GPa); alternative EoS forms provided poorer agreement in this regime (Figure S2). Beyond 10.2 GPa, the experimental void volumes progressively fall below the extrapolated fit, with the deviation becoming pronounced near 26–27 GPa, indicating that interlayer free space is effectively exhausted and further compression must be accommodated by molecular reorganization rather than continued void reduction. This is reflected in the plot of molecular volume versus pressure (Figure 4d), which shows a distinct change in gradient between 26.6 and 27.7 GPa. This change in slope coincides with conformational adjustments described below.

### 3.4 Structural Changes in Ribavirin V1 on Compression

Ribavirin is conformationally flexible, and its structure responds anisotropically to compression.^22^ In **V1**, three compression regimes are evident, defined by inflection points near 10.9 and 26.6 GPa in the axial compressibility and molecular volume. Compression proceeds initially through interlayer void collapse (0–10.9 GPa), then through progressive layer flattening and redistribution of lattice strain (10.9–26.6 GPa) and finally through predominantly intramolecular conformational accommodation once residual void space is nearly exhausted (>26.6 GPa). Packing evolution is quantified using the ‘corrugation angle’, defined as the angle between least-squares planes fitted through molecules *F*/*F*′ and *D*′/*G*′ in the top molecular layer of the first coordination sphere (Figure 5a and Figure S3). Conformational changes are illustrated by the structural overlay and monitored through selected torsion angles (Figure 5b and Figure S5) and ribofuranosyl puckering parameters (Figure 6); several torsions and the puckering descriptors exhibit slope changes near the same pressures.

**Figure 5:**
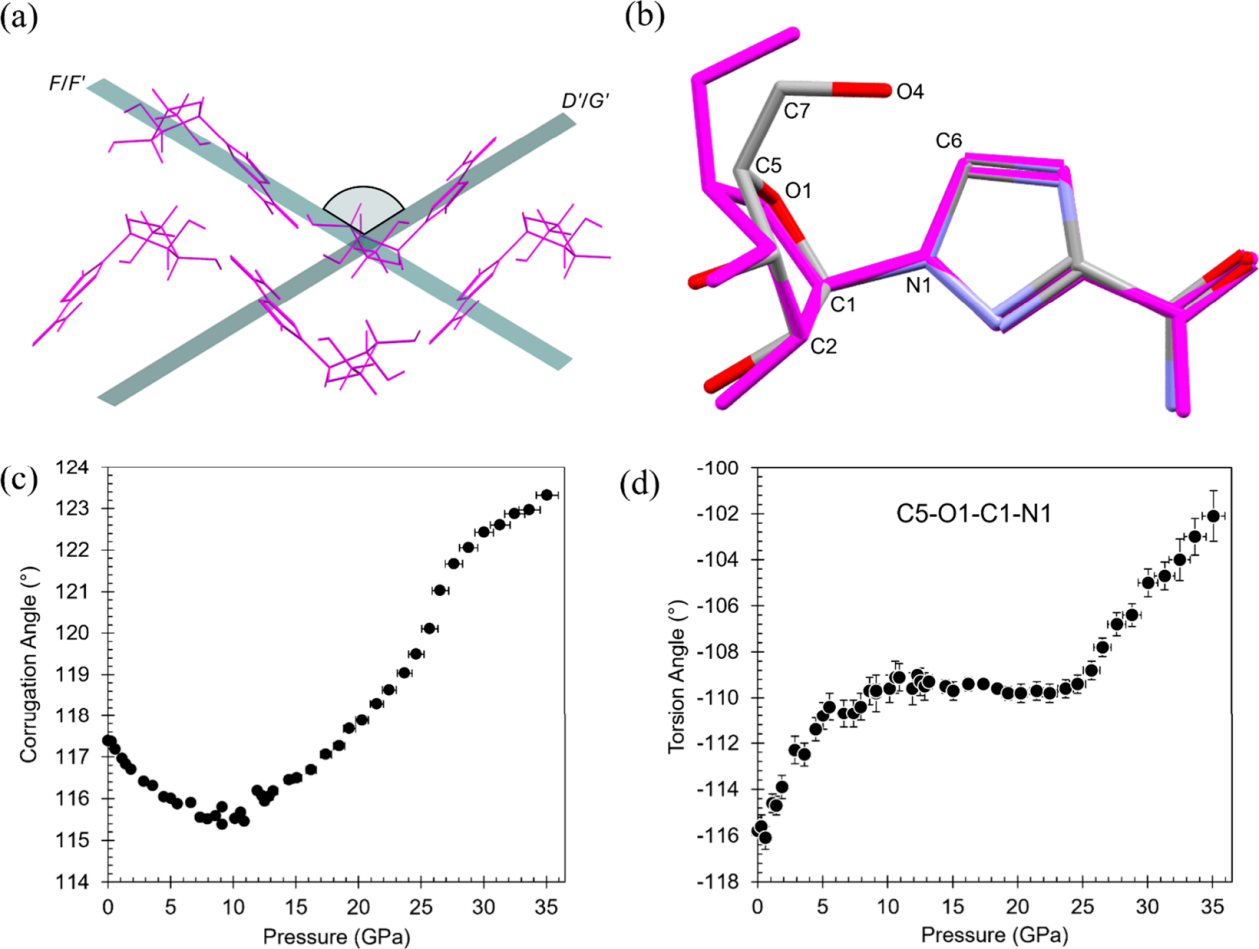
Structural evolution of ribavirin **V1** under compression. (a) Definition of the corrugation angle between least-squares planes fitted through molecules *F*/*F*′ and *D*′/*G*′ within the top molecular layer of the first coordination sphere. (b) Overlay of the ambient-pressure (magenta) and 35.1 GPa (colored by element) structures highlighting conformational changes in the hydroxymethyl and ribofuranosyl groups at the glycosidic bond. Pressure dependence of (c) the corrugation angle and (d) the C5–O1–C1–N1 torsion angle.

**Figure 6:**
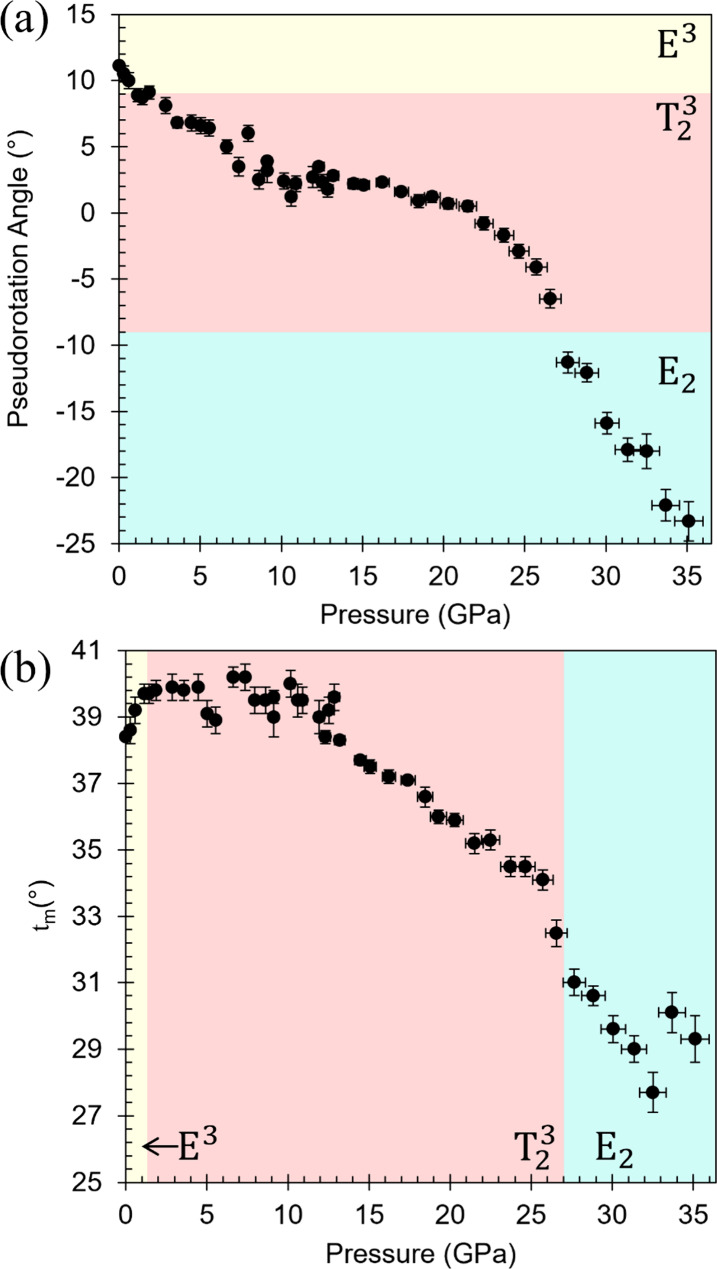
(a) Pseudorotation angle *P* and (b) puckering amplitude τ_m_, of the ribofuranosyl ring in ribavirin **V1** as a function of pressure. *P* Angle values are shown in their wrapped form to preserve continuity near the 0°/360° boundary. Regions are shaded according to dominant ring conformation.

In the initial compression regime (0–10.9 GPa), pressure is accommodated primarily through collapse of interlayer void space, with an 11.9% reduction in the *b*-axis and a 76.6% decrease in void volume. Over this range, the molecular layers contract with little internal rearrangement, and the corrugation angle decreases slightly from 117.38(2)° to 115.52(5)° (Figure 5c), consistent with a modest increase in layer puckering. Although the ribofuranosyl ring undergoes an early E^3^ → T_2_^3^ puckering transition at 1.1 GPa, there is no significant effect on the unit cell parameters or packing topology. Compression in this regime is dominated by interlayer void collapse with minimal internal reorganization.

Between 10.9 and 26.6 GPa, compression proceeds under substantially reduced free volume, with the void volume per molecule decreasing from 11.67(4) Å^3^ at 10.9 GPa to 2.83(1) Å^3^ at 26.6 GPa. Over this range, the contribution of simple void collapse diminishes, and increasing intermolecular strain within the hydrogen-bonded layers is accommodated through coordinated intramolecular torsional adjustments. Several torsion angles exhibit slope changes near 11 GPa (Figure S5). A representative example is the C5–O1–C1–N1 torsion, which undergoes a rapid initial adjustment below 6 GPa, remains approximately constant between 11 and 25 GPa, and increases again above 25 GPa (Figure 5d). Concomitantly, the corrugation angle increases toward coplanarity (180°) from 115.45(5)° to 121.02(4)°, indicating progressive flattening of the molecular layers and accounting for the expansion of the *c*-axis beyond 10.9 GPa while retaining symmetry and preserving the hydrogen-bonded network, as illustrated in Figure S4.

By 27.7 GPa, the void space is nearly exhausted (94.8% collapse relative to ambient pressure). A small but statistically significant decrease in *V*_cell_/*Z* (Δ*V* = – 0.76 Å^3^ per molecule) occurs between 26.6 and 27.7 GPa, coincident with reorientation of the hydroxymethyl group across the upper face of the ribofuranosyl ring and a T_2_^3^ → E_2_ puckering transition. The hydroxymethyl group reorientation shortens the molecular length approximately parallel to the C2–C5 axis. This is accompanied by continued increase of the C5–O1–C1–N1 torsion across the 26–35 GPa interval, consistent with progressive adjustment at the glycosidic bond. The associated molecular volume reduction corresponds to a *p*Δ*V* contribution of approximately – 12 kJ mol^–1^ at 27.7 GPa, favoring the adoption of the more compact molecular geometry. Additional torsional changes occur across the molecule, with several angles exhibiting slope changes near this pressure. With residual void space largely eliminated, further compression continues predominantly through intramolecular conformational adjustment. This contributes to the expansion of the *c*-axis, which approaches a plateau above 30 GPa, while layer flattening persists at a reduced rate.

Conformational changes within the ribofuranosyl ring exhibit slope changes at pressures coincident with those identified for packing and torsional evolution. The pseudorotation angle, *P*, shows slope changes near 11 and 27 GPa (Figure 6a). Two puckering transitions are observed: an early E^3^ →T_2_^3^ transition at 1.1 GPa (discussed above) and a T_2_^3^ → E_2_ at 27.7 GPa. The latter coincides with hydroxymethyl reorientation. The puckering amplitude, τ_m_, remains relatively constant below 12 GPa (39–40°), then decreases progressively to 34.1(3)° at 25.7 GPa followed by a pronounced drop to 32.5(4)° at 26.6 GPa and reaching a minimum of 27.7(6)° at 32.5 GPa (Figure 6b). The overall decrease in τ_m_ on compression indicates progressive flattening of the ribofuranosyl ring, which is evident in the structural overlay of the ambient and 35.1 GPa models (Figure 5b).

Comparison with the previously reported **V2** compression series points to differences in hydrogen-bonded packing as a key factor in how ribavirin accommodates pressure. In **V2**, pressure-induced changes in ribofuranosyl puckering are associated with the high-pressure forms **V3**–**V5**, which occupy separate regions of pseudorotation space.^22^ In **V1**, the corresponding puckering changes occur along a continuous compression pathway while the *P*2_1_2_1_2_1_ symmetry and principal hydrogen-bonded topology are retained (Figure S6). The differing responses indicate that strain accommodation depends on how conformational flexibility is coupled to crystal packing. The ambient-pressure hydrogen-bonded packing arrangements of **V1** and **V2** differ significantly (Figure S7), imposing different structural constraints on the ribofuranosyl group during compression. In **V1**, the corrugated H-bonded layers are retained, allowing strain to be accommodated by interlayer void collapse and progressive layer flattening before higher-pressure reorientation of the hydroxymethyl group. In **V2**, ribofuranosyl conformational changes are coupled to changes in the supramolecular hydrogen-bonded arrangement between high-pressure forms. The energetic response of the individual molecule–molecule interactions in **V1** is discussed below.

### 3.5 Intermolecular Interaction Energies on Compression in Ribavirin V1

The evolution of the seven symmetry-unique intermolecular interactions (*A*–*G*) within the first molecular coordination sphere was analyzed as a function of pressure using the PIXEL method. All *A*–*G* dimers were benchmarked against SAPT0 calculations at selected pressures (Table S4), and the distance dependence of the total interaction energies is compared in Figure S8. Although SAPT0 yields smaller absolute interaction energies, both approaches reproduce the same ambient ordering and closely similar pressure-dependent trends across the compression regime. Total interaction energies are plotted as a function of pressure in Figure 7, and listed in Table S5.

**Figure 7:**
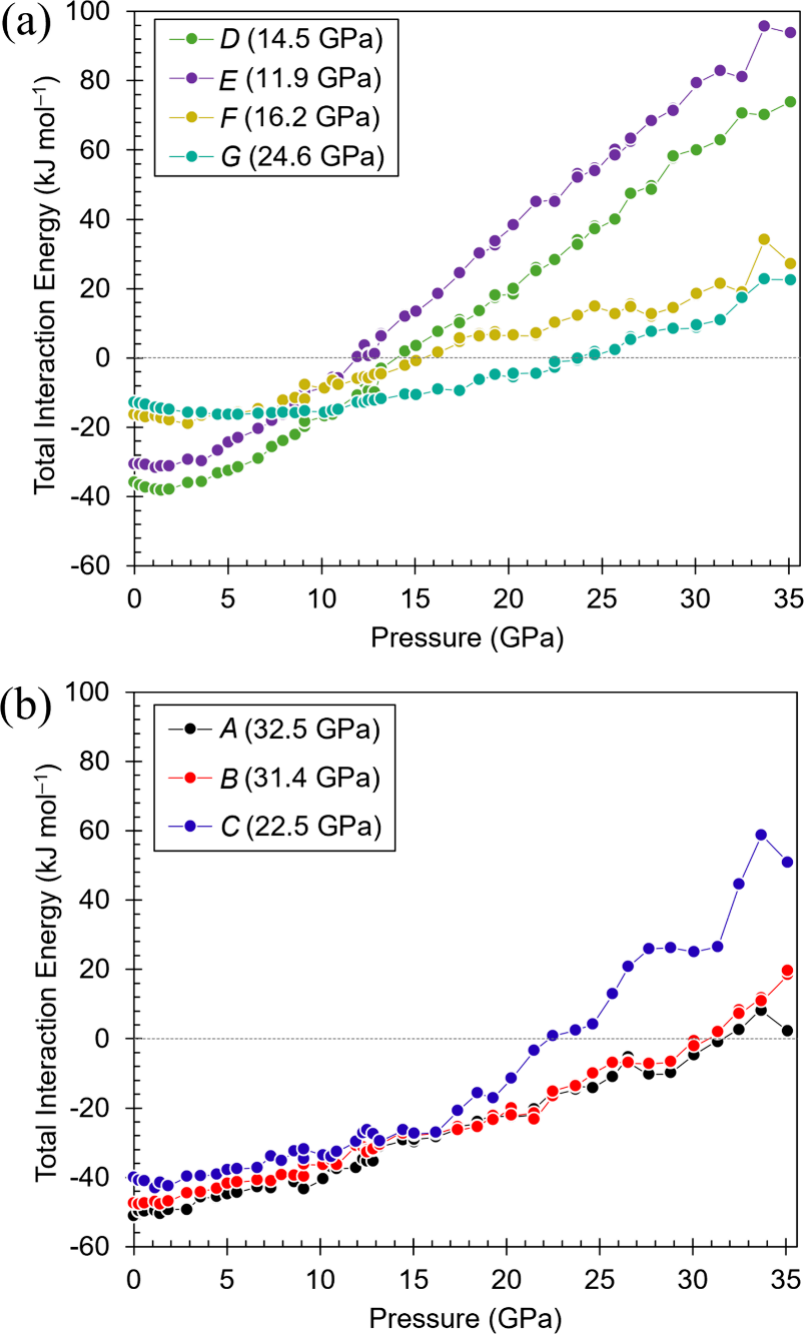
Total interaction energies calculated by PIXEL for intermolecular contacts in the first coordination sphere of ribavirin **V1** as a function of pressure. (a) Interactions *D*–*G*; (b) Interactions *A*–*C*. The pressures at which each interaction becomes destabilizing (positive) are indicated in parentheses in the legends.

Total interaction energies become progressively less favorable (more positive) with increasing pressure. The increase is most pronounced for the interlayer interactions (Figure 7a). The first sign inversion occurs between 10.9 and 11.9 GPa, with the energy of interaction *E* becoming positive at 11.9 GPa. This pressure coincides with the upper boundary of the void-collapse regime identified from the equation-of-state analysis. Interlayer interactions are thus the first to become net repulsive as free volume is depleted. Further sign inversions in the interaction energies occur for *D* (∼15 GPa) and *F* (∼16 GPa), followed by *C* and *G* within the ∼ 22–25 GPa interval. The strongest hydrogen-bonded contacts, *B* and *A*, remain stabilizing until 31.4 and 32.5 GPa, respectively (Figure 7b).

Energy decomposition (Figure S9) shows that Coulombic and polarization contributions increase in magnitude as contact distances shorten. Dispersion likewise becomes more stabilizing on compression. However, the exchange-repulsion term increases more steeply and ultimately dominates the total interaction energy. The sign inversions therefore arise from the steep increase in exchange-repulsion under compression rather than the loss of attractive contributions.

As seen above, by 26–27 GPa, interlayer void space is largely eliminated and intramolecular adjustment becomes prominent. At this stage, *E*, *D*, *F*, *C* and *G* are repulsion-dominated, while *A* and *B* remain stabilizing (≈ – 6 to – 7 kJ mol^–1^ at 26.6 GPa). Minor deviations from monotonic behavior in interactions *A*–*C* (Figure 7b) coincide with the hydroxymethyl reorientation and the T_2_^3^ → E_2_ puckering transition.

Hydrogen bonds within interactions *A*–*C* remain topologically intact to 35.1 GPa, although additional contacts emerge on compression in *A* (N4···O2 above 19.3 GPa) and *B* (O3···O5 between ∼ 9–11 GPa); interaction *C* retains the same hydrogen-bond motif throughout (Figure S10). Donor–acceptor distances (D···A) shorten by up to ∼ 0.4 Å (e.g., N4···O3:2.99 → 2.61 Å; O4···N2:2.92 → 2.48 Å), and several contacts adopt geometries that are statistically uncommon within the CSD distributions (Table S6) although N4···O4 (2.98 Å, ∠ = 158.5°) remains within typical ranges. For O3H3···N3 in *B*/*B′*, shortening of the donor–acceptor distance is accompanied by angular distortion, D–H···A, decreasing from 145° to 123° between ambient pressure and 35.1 GPa.

At 35.1 GPa, all interaction energies calculated using PIXEL are marginally to strongly positive. The magnitude of destabilization varies substantially: *A* remains close to zero (≈ + 2 kJ mol^–1^), whereas *E* reaches ≈ + 94 kJ mol^–1^. The ordering at high pressure does not mirror the ambient ranking of interaction strengths but reflects how rapidly repulsion increases for each contact as separations are reduced. The energy–pressure profiles therefore quantify the relative resistance of each interaction to compression.

## 4 Conclusions

High-pressure, high-temperature recrystallization in the diamond anvil cell provides an effective route to obtaining single crystals of the elusive **V1** polymorph of ribavirin and may offer a general strategy for accessing dense or metastable molecular phases that are inaccessible by conventional crystallization techniques. The structure of ribavirin **V1** has been refined to 35.1 GPa, placing it among the few hydrogen-bonded molecular crystals characterized well beyond 10 GPa by single-crystal X-ray diffraction.

Ribavirin is a relatively large molecule with a high degree of conformational flexibility in the ribofuranosyl ring. In our previous study of the **V2** form, a series of phase transitions up to 7 GPa was coupled to discontinuous conformational changes in the ribofuranosyl ring. While there are no pressure-driven phase transitions in form **V1**, the flexibility of the ribofuranosyl ring is an important mechanism for accommodating pressure above ∼ 11 GPa, but particularly above ∼ 27 GPa when the intermolecular void space is largely exhausted.

The pressure reached in the present study is among the highest attained for a hydrogen-bonded organic material, but it is notable that during compression of the H-bonded sheets, the hydrogen-bonded topology is preserved and, even at 35 GPa, the H-bonded N···O distances remain above 2.4 Å. To be sure, this is at the low end of N···O interactions in the CSD, but it is not without precedent even at much lower pressures. N···O distances in cocrystals such as GADGUN16 (pentachlorophenol:4-methylpyridine)^56^ and HUSTOE31 (butanedioic acid:4,4’-(ethane-1,2-diyl)­dipyridine)^57^ both reach 2.43 Å at pressures almost an order of magnitude lower (∼3.7 GPa) than in the present study, and even at ambient pressure, the crystal structure YAHJIC01 ((E)-1,2-bis­(pyridin-2-yl)­ethene: pyridine-2,4-dicarboxylic acid) exhibits a value of 2.47 Å.^58^ The hydrogen bond network in melamine is similarly robust.^14^

Phase transitions involving rearrangement of discrete molecular packing appear to be rare beyond 20 GPa. Where observed, such transitions, as in fluoroaromatic cocrystals,^10^ benzene, and pyridine, are often associated with cross-linking and polymerization.^59,60^ A transition from monoclinic to triclinic symmetry at 36.1 GPa has been claimed for melamine at 30 GPa in Ne and 36 GPa in He, though no crystal structure of the high-pressure phase appears to be available.^14^ The approach to plateau behavior in **V1** in both lattice parameters and conformational metrics at the highest pressures studied suggests that the principal modes of strain accommodation become increasingly limited above ∼ 30 GPa. Beyond this regime, further densification would likely require qualitatively different structural responses.

## Supplementary Material



## Notes

Supplementary crystallographic data, figures, tables and analyses; compiled high-pressure CSD data set; crystallographic information files (PDF)
