# Supplementary material for: Compression of Ribavirin to 35 GPa
Source: Cryst Growth Des. 2026 May 14;26(11):4302–12. doi: 10.1021/acs.cgd.6c00376 (PMC13237730; doi:10.1021/acs.cgd.6c00376)
Supplement: Supplementary file 1 [file cg6c00376_si_001.pdf]

Supplementary Information

Compression of Ribavirin to 35 GPa

Bhaskar Tiwari[1,2], Simon Parsons[1], Nico Giordano[2*]

1. Centre for Science at Extreme Conditions, School of Chemistry, The University of Edinburgh, EH9 3FJ, Edinburgh, U.K.

2. Deutsches Elektronen-Synchrotron DESY, Notkestr. 85, 22607 Hamburg, Germany

Search of the Cambridge Structural Database

A dataset was compiled from the Cambridge Structural Database (CSD, version 6.0, August 2025 release), accessed 29 October 2025, using the high-pressure subset in ConQuest.[1]

The search was restricted to entries determined by single-crystal X-ray diffraction (SC-XRD) that contain full three-dimensional atomic coordinates, include only non-metal atoms, and feature at least one C–X bond (X = any element). Structures containing ionic species or polymeric networks were excluded to retain only discrete molecular systems. No limits were applied to refinement quality indicators. Additional recent structures not yet incorporated into the database were manually added from the primary literature.

The resulting dataset comprises 2114 entries (2045 from the CSD and 69 recent literature additions). Within the compiled dataset, most SC-XRD entries were collected below 10 GPa (1959 of 2114 entries, 93%), and only 57 entries (2.7% of the dataset) extend beyond 20 GPa (**Fig S1. (a)**). Reported refinement quality as a function of pressure is shown in **Fig. S1(b)**. Representative examples refined above 10 GPa are listed in **Table S1**, while the complete dataset is provided in the accompanying Excel file (*CSD_high_pressure_dataset.xlsx*) which contains the raw ConQuest export and additional literature-derived structures not yet in the CSD.

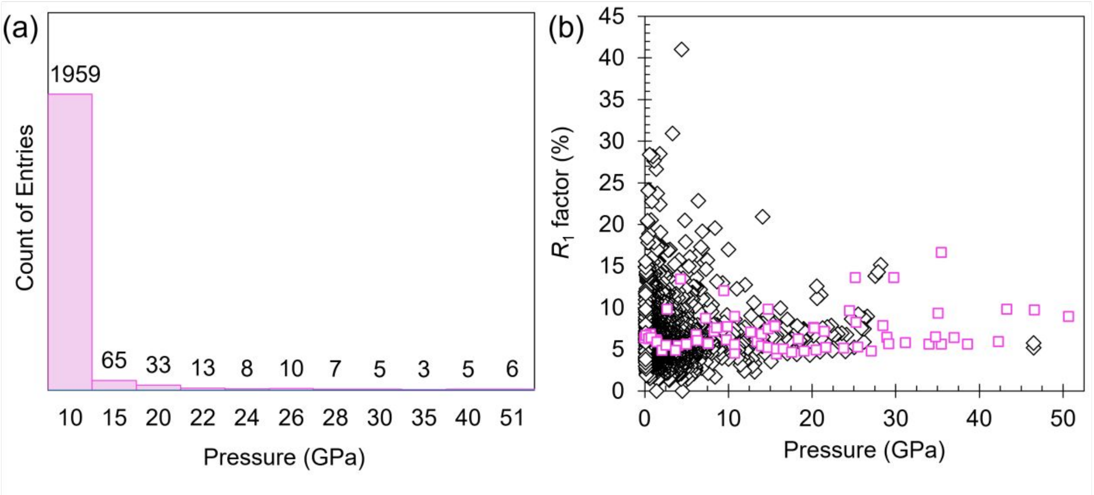

Figure S1 (a) *Binned distribution of reported measurement pressures for single-crystal X-ray diffraction (SC-XRD) structures in the compiled high-pressure dataset (CSD v6.0, August 2025 release, accessed 29 October 2025, together with recent literature additions not yet indexed in the CSD). Frequency of entries within defined pressure bins; x-axis values indicate the upper limit of each bin (e.g., "10 GPa" corresponds to pressures ≤10 GPa, "15 GPa" to 10–15 GPa, etc.), and bars are labelled with the number of structures in each interval. (b) Reported R_1 values as a function of measurement pressure for all entries in the same dataset. Black diamonds represent structures retrieved directly from the CSD, and pink squares denote additional literature structures not yet incorporated into the database.*

Table S1 *Summary of high-pressure single-crystal X-ray diffraction studies of organic molecular systems above 10 GPa.*

Reference	Chemical Formula Moiety	Chemical Name Systematic	Temperature (K)	Pressure (GPa)	R_1-factor (%)	Notes
2	C12 H10 N2	diphenyldiazene	298	28.21	15.17	Polymerizes above 28 GPa. Study continues to 33 GPa.
3	C20 H12	benzo[pqr]tetraphene	293	27.9	14.27	
4	C6 H6 B1 N1 O6	(Nitrilotriacetato-N,O,O',O'')-boron	298	16.2	9.95	
5	C14 H10,C10 F8	anthracene octafluoronaphthalene	293	22.56	5.15	Co-crystal. Polymerizes above 25 GPa. Study continues to 29.2 GPa.
5	C10 H8,C10 F8	naphthalene octafluoronaphthalene	293	18.92	7.34	Co-crystal. Polymerizes above 19 GPa. Study continues to 25.6 GPa
6	C7 H5 N1 O1	4-hydroxybenzonitrile	293	14.14	6.5	Forms a He co-crystal above 15 GPa (below).
6	C7 H5 N1 O1:He1	4-hydroxybenzonitrile helium	293	26.19	7.46	Co-crystal.
7	C6 H6 O2,0.33(C2 H3 N1)	benzene-1,4-diol acetonitrile clathrate	259	14.1	20.94	Lab source data. Temperature value may be typo. Rest of study at 295 K.
8	C4 H9 N1 O3	L-threonine	298	22.31	7.85	
9	C6 H12 N4	1,3,5,7-tetraazatricyclo [3.3.1.1^{3,7}]decane	293	13.13	2.29	SC-XRD search returns 0.34–13.13 GPa but structural data to 20 GPa (perhaps PXRD; see paper)

Table 1 continued *Summary of high-pressure single-crystal X-ray diffraction studies of organic molecular systems above 10 GPa.*

Reference	Chemical Formula Moiety	Chemical Name Systematic	Temperature (K)	Pressure (GPa)	R_1-factor (%)	Notes
10	C1 F4	tetrafluoromethane	293	46.5	5.76/5.21	Simple molecular system. Polymorphs. Multiple crystals measured in same DAC
11	C48 H20	benzo[1,2,3-bc:4,5,6-b'c']dicoronene	298	26.2	5.9	
12	C16 H10	pyrene	298	35.5	16.6	Structure undergoing enhancement. Manual entry; not part of ConQuest search results
13	C10 H8	naphthalene	298	50.7	8.9	Manual entry; not part of ConQuest search results
13	C14 H10	anthracene	298	42.3	5.9	Manual entry; not part of ConQuest search results
14	C14 H14	1,2-diphenylethane	298	37	6.35	Manual entry; not part of ConQuest search results

Summary of Crystallographic Data

Table S2 *Summary of crystal data and refinement parameters of ribavirin **V1** at ambient and high pressure.*

Pressure (GPa)	0.0	0.3	0.6	1.1	1.4	1.9
Phase	V1	V1	V1	V1	V1	V1
Crystal Data						
a, b, c (Å)	7.5198 (8), 8.8235 (2), 14.8847 (6)	7.4961 (14), 8.7572 (10), 14.8433 (9)	7.4798 (19), 8.7084 (14), 14.8060 (12)	7.4249 (9), 8.5834 (6), 14.7637 (6)	7.4022 (10), 8.5351 (7), 14.7248 (6)	7.3663 (8), 8.4638 (5), 14.6981 (5)
V (Å³)	987.61 (11)	974.4 (2)	964.4 (3)	940.90 (14)	930.29 (15)	916.38 (12)
Z	4	4	4	4	4	4
Data Collection						
No. of measured, independent and observed [$I > 2\sigma(I)$] reflections	2380, 1188, 1138	2153, 1182, 972	2239, 1183, 968	2085, 1382, 1228	2073, 1348, 1193	1980, 1307, 1148
R_{int}	0.015	0.048	0.045	0.041	0.044	0.045
Refinement						
$R[F^2 > 2\sigma(F^2)]$, $wR(F^2)$, S	0.027, 0.071, 1.11	0.050, 0.147, 1.08	0.049, 0.131, 1.09	0.049, 0.145, 1.09	0.050, 0.156, 0.98	0.050, 0.161, 1.02

Table S2 continued *Summary of crystal data and refinement parameters of ribavirin* ***V1*** *at high pressure.*

Pressure (GPa)	2.9	3.6	4.5	5.0	5.5	6.6
Phase	V1	V1	V1	V1	V1	V1
Crystal Data						
a, b, c (Å)	7.2801 (6), 8.3106 (4), 14.6084 (5)	7.2391 (6), 8.2454 (4), 14.5665 (5)	7.1833 (7), 8.1584 (5), 14.4990 (6)	7.1504 (6), 8.1138 (4), 14.4756 (5)	7.1294 (5), 8.0849 (4), 14.4622 (5)	7.0769 (7), 8.0093 (5), 14.4137 (7)
V ($Å^3$)	883.84 (9)	869.46 (9)	849.70 (10)	839.83 (9)	833.61 (8)	816.98 (10)
Z	4	4	4	4	4	4
Data Collection						
No. of measured, independent and observed [$I > 2\sigma(I)$] reflections	2073, 1348, 1193	1980, 1307, 1148	1748, 1229, 1076	1825, 1243, 1146	1790, 1236, 1133	1743, 1207, 1065
R_{int}	0.044	0.045	0.055	0.047	0.047	0.043
Refinement						
$R[F^2 > 2\sigma(F^2)]$, $wR(F^2)$, S	0.050, 0.156, 0.98	0.050, 0.161, 1.02	0.059, 0.178, 1.00	0.053, 0.155, 1.13	0.051, 0.144, 1.09	0.048, 0.141, 0.99

Table S2 continued *Summary of crystal data and refinement parameters of ribavirin* ***V1*** *at high pressure.*

Pressure (GPa)	**7.4**	**8.0**	**8.6**	**9.1**	**9.1**	**10.2**
Phase	V1	V1	V1	V1	V1	V1
Crystal Data						
a, *b*, *c* (Å)	7.0448 (7), 7.9677 (5), 14.3983 (6)	7.0100 (5), 7.9232 (4), 14.3704 (5)	6.9851 (8), 7.8811 (5), 14.3493 (6)	6.9789 (10), 7.8622 (7), 14.3585 (8)	6.9755 (7), 7.8231 (3), 14.3428 (6)	6.9309 (7), 7.8128 (5), 14.3156 (6)
V (Å^3)	808.19 (10)	798.16 (8)	789.93 (11)	787.84 (14)	782.69 (9)	775.19 (10)
Z	4	4	4	4	4	4
Data Collection						
No. of measured, independent and observed [$I > 2\sigma(I)$] reflections	1802, 1236, 1081	1811, 1227, 1098	1834, 1224, 1053	1482, 1078, 824	1410, 864, 833	1755, 1173, 1020
R_{int}	0.046	0.042	0.057	0.063	0.031	0.048
Refinement						
$R[F^2 > 2\sigma(F^2)]$, $wR(F^2)$, S	0.061, 0.195, 1.15	0.053, 0.169, 1.06	0.069, 0.222, 1.13	0.084, 0.259, 1.10	0.040, 0.107, 1.07	0.060, 0.181, 1.10

Table S2 continued *Summary of crystal data and refinement parameters of ribavirin* ***V1*** *at high pressure.*

Pressure (GPa)	**10.6**	**10.9**	**11.9**	**12.3**	**12.5**	**12.9**
Phase	V1	V1	V1	V1	V1	V1
Crystal Data						
a, b, c (Å)	6.9105 (8), 7.7893 (6), 14.3191 (8)	6.8978 (7), 7.7769 (5), 14.3042 (6)	6.8690 (7), 7.6809 (5), 14.3306 (8)	6.8588 (5), 7.64537 (18), 14.3288 (4)	6.8426 (8), 7.6757 (7), 14.3225 (9)	6.8389 (9), 7.6661 (7), 14.3307 (10)
V (Å^3)	770.77 (12)	767.33 (10)	756.08 (10)	751.37 (6)	752.24 (12)	751.33 (13)
Z	4	4	4	4	4	4
Data Collection						
No. of measured, independent and observed [$I > 2\sigma(I)$] reflections	1611, 1126, 914	1781, 1180, 1035	1473, 1039, 833	1740, 1126, 1107	1719, 1143, 975	1588, 1087, 879
R_{int}	0.051	0.052	0.055	0.022	0.038	0.044
Refinement						
$R[F^2 > 2\sigma(F^2)]$, $wR(F^2)$, S	0.066, 0.225, 1.14	0.057, 0.169, 1.14	0.068, 0.207, 1.10	0.032, 0.088, 1.10	0.054, 0.163, 1.10	0.055, 0.169, 1.08

Table S2 continued *Summary of crystal data and refinement parameters of ribavirin* ***V1*** *at high pressure.*

Pressure (GPa)	13.2	14.5	15.1	16.2	17.4	18.5
Phase	V1	V1	V1	V1	V1	V1
Crystal Data						
a, b, c (Å)	6.8287 (5), 7.5993 (2), 14.3326 (5)	6.7849 (6), 7.5292 (2), 14.3554 (5)	6.7698 (8), 7.5096 (3), 14.3577 (7)	6.7300 (7), 7.4538 (3), 14.3702 (6)	6.6925 (6), 7.4017 (2), 14.4019 (5)	6.6493 (8), 7.3619 (3), 14.4212 (7)
V (Å³)	743.76 (6)	733.35 (7)	729.92 (9)	720.87 (8)	713.41 (7)	705.94 (10)
Z	4	4	4	4	4	4
Data Collection						
No. of measured, independent and observed [$I > 2\sigma(I)$] reflections	1675, 1092, 1069	1696, 1074, 1057	1856, 1076, 1059	1811, 1052, 1029	1789, 1041, 1016	1747, 1046, 1007
R_{int}	0.019	0.021	0.029	0.018	0.024	0.028
Refinement						
$R[F^2 > 2\sigma(F^2)]$, $wR(F^2)$, S	0.028, 0.073, 1.09	0.032, 0.080, 1.02	0.033, 0.086, 1.12	0.030, 0.080, 1.13	0.031, 0.079, 1.07	0.038, 0.114, 1.20

Table S2 continued *Summary of crystal data and refinement parameters of ribavirin* ***V1*** *at high pressure.*

Pressure (GPa)	19.3	20.3	21.5	22.5	23.7	24.6
Phase	V1	V1	V1	V1	V1	V1
Crystal Data						
a, b, c (Å)	6.6278 (7), 7.3138 (3), 14.4618 (6)	6.5913 (9), 7.2836 (4), 14.4829 (9)	6.5569 (9), 7.2385 (3), 14.5059 (6)	6.5182 (13), 7.2108 (5), 14.5413 (12)	6.4626 (18), 7.1698 (7), 14.5793 (16)	6.4294 (19), 7.1408 (7), 14.6225 (17)
V (Å³)	701.03 (8)	695.30 (11)	688.49 (10)	683.47 (16)	675.5 (2)	671.3 (2)
Z	4	4	4	4	4	4
Data Collection						
No. of measured, independent and observed [$I > 2\sigma(I)$] reflections	1589, 1017, 981	1595, 1017, 977	1619, 1011, 958	1677, 1000, 945	1624, 975, 910	1559, 966, 869
R_{int}	0.024	0.022	0.031	0.030	0.036	0.039
Refinement						
$R[F^2 > 2\sigma(F^2)]$, $wR(F^2)$, S	0.031, 0.085, 1.15	0.032, 0.086, 0.96	0.037, 0.100, 1.12	0.039, 0.107, 1.12	0.043, 0.111, 1.08	0.049, 0.133, 1.10

Table S2 continued *Summary of crystal data and refinement parameters of ribavirin* ***V1*** *at high pressure.*

Pressure (GPa)	25.7	26.6	27.7	28.8	30.1	31.4
Phase	V1	V1	V1	V1	V1	V1
Crystal Data						
a, b, c (Å)	6.391 (2), 7.1077 (7), 14.6735 (18)	6.341 (2), 7.0699 (8), 14.729 (2)	6.307 (2), 7.0405 (8), 14.759 (2)	6.278 (3), 7.0020 (9), 14.785 (2)	6.234 (3), 6.9771 (11), 14.803 (3)	6.204 (4), 6.9646 (12), 14.805 (3)
V (Å³)	666.6 (2)	660.3 (3)	655.3 (3)	649.9 (3)	643.8 (4)	639.7 (4)
Z	4	4	4	4	4	4
Data Collection						
No. of measured, independent and observed [$I > 2\sigma(I)$] reflections	1502, 952, 858	1584, 959, 845	1516, 935, 805	1532, 921, 791	1500, 918, 783	1456, 907, 756
R_{int}	0.037	0.041	0.036	0.031	0.044	0.043
Refinement						
$R[F^2 > 2\sigma(F^2)]$, $wR(F^2)$, S	0.047, 0.134, 1.09	0.048, 0.132, 1.14	0.052, 0.148, 1.10	0.047, 0.133, 1.10	0.054, 0.148, 1.05	0.057, 0.163, 1.06

Table S2 continued *Summary of crystal data and refinement parameters of ribavirin* ***V1*** *at high pressure.*

Pressure (GPa)	32.5	33.7	35.1
Phase	V1	V1	V1
Crystal Data			
a, b, c (Å)	6.156 (5), 6.9524 (14), 14.808 (3)	6.145 (4), 6.9215 (15), 14.824 (4)	6.093 (5), 6.9256 (18), 14.811 (4)
V (Å³)	633.7 (5)	630.5 (5)	625.0 (6)
Z	4	4	4
Data Collection			
No. of measured, independent and observed [$I > 2\sigma(I)$] reflections	1187, 771, 597	1236, 838, 676	1254, 792, 590
R_{int}	0.049	0.046	0.050
Refinement			
$R[F^2 > 2\sigma(F^2)]$, $wR(F^2)$, S	0.070, 0.217, 1.13	0.076, 0.225, 1.14	0.076, 0.238, 1.15

Volume Analysis

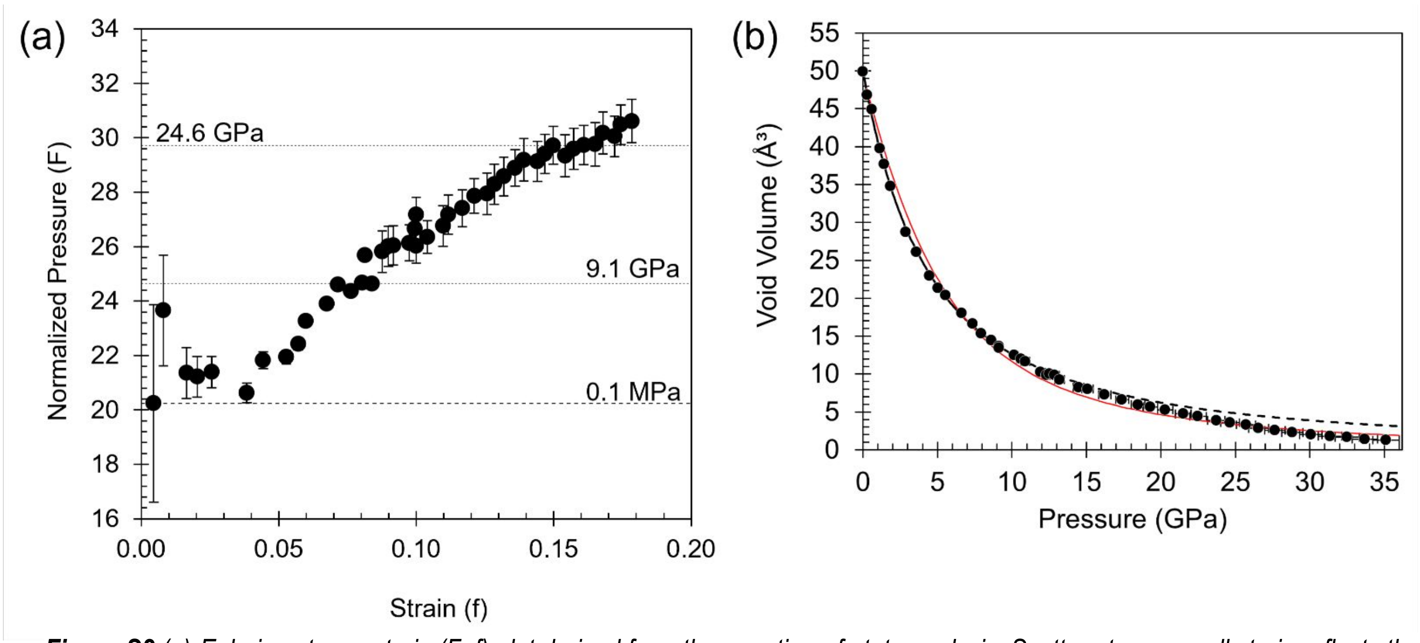

Figure S2 *(a) Eulerian stress–strain (F–f) plot derived from the equation-of-state analysis. Scatter at very small strain reflects the limited sensitivity of the F–f linearization near $V \approx V_0$. Changes in slope near ~10 and ~25 GPa are consistent with regime-dependent compressibility. (b) Void volume as a function of pressure with Vinet equation-of-state fits. The fit restricted to 0–10.2 GPa is shown as a solid black line, with its extrapolation as a dashed black line. A fit over the full pressure range (red line) fails to reproduce the observed curvature and results in systematic deviations, demonstrating that a single equation of state does not adequately describe the full compression behavior.*

Crystal Packing & Corrugated Layers

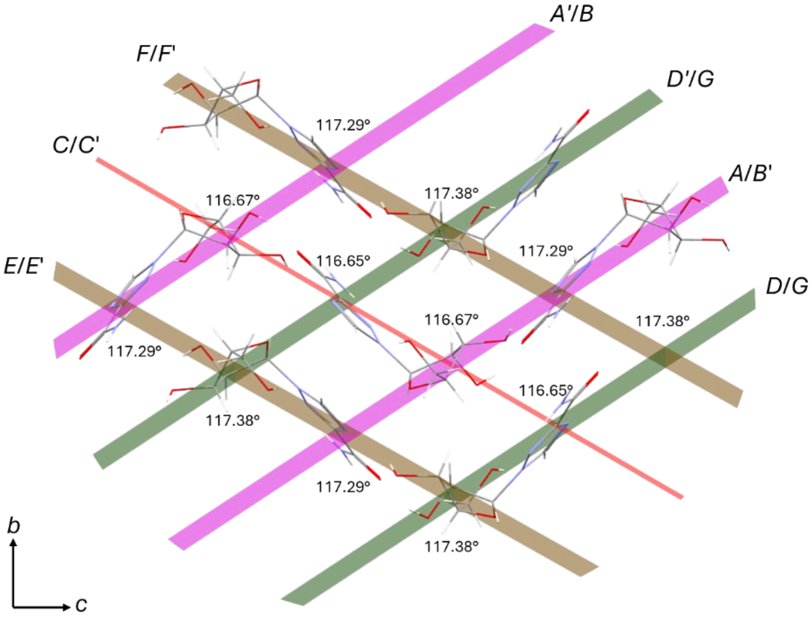

Figure S3 *Comparison of corrugation angles obtained using alternative symmetry-related plane definitions within the first coordination sphere. Angles are reported as the supplementary angle (180° − φ) between least-squares planes fitted through all atoms of the selected molecules. The values discussed in the main text correspond to the F/F' vs D'/G' definition obtained from SHELX. Alternative plane selections yield corrugation angles within 1° of this value and exhibit identical pressure-dependent behaviour (see Table S3).*

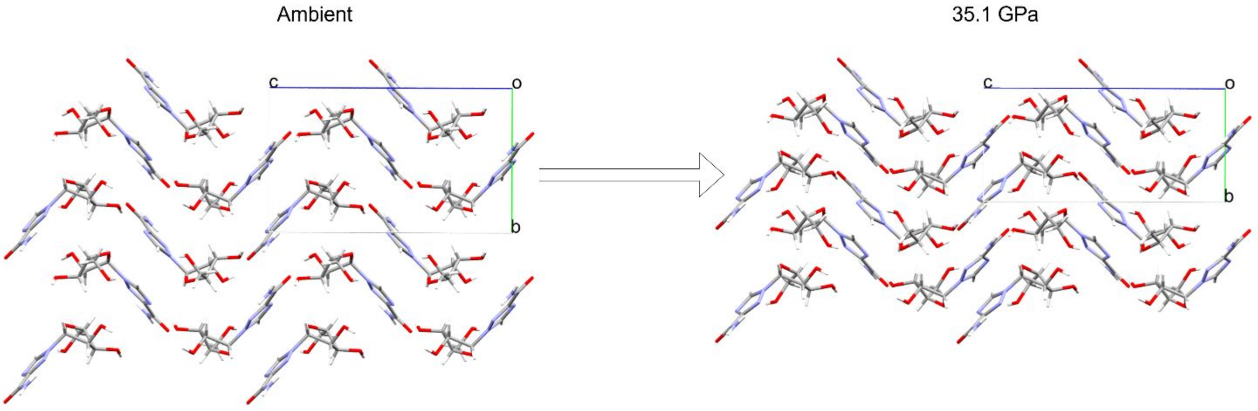

Figure S4 *Comparison of molecular packing in ribavirin **V1** at ambient pressure and 35.1 GPa. The corrugated hydrogen-bonded layers stack along b and remain topologically preserved over the full compression range.*

Table S3 *Corrugation angles obtained using alternative symmetry-related plane definitions within the first coordination sphere at 0 and 35.1 GPa. Angles are reported as the supplementary angle (180° − φ) between least-squares planes fitted through all atoms of the selected molecules. Variations between alternative definitions are ≤1° at both pressures.*

Plane definition	0 GPa (°)	35.1 GPa (°)
F/*F*' vs *D*'/*G*	117.38	123.28
F/*F*' vs *A*'/*B*	117.29	123.2
F/*F*' vs *A*/*B*'	117.29	123.2
F/*F*' vs *D*/*G*	117.38	123.28
C/*C*' vs *D*'/*G*	116.65	122.79
C/*C*' vs *A*'/*B*	116.67	122.81
C/*C*' vs *A*/*B*'	116.67	122.81
C/*C*' vs *D*/*G*	116.65	122.79
E/*E*' vs *D*'/*G*	117.38	123.28
E/*E*' vs *A*'/*B*	117.29	123.2
E/*E*' vs *A*/*B*'	117.29	123.2
E/*E*' vs *D*/*G*	117.38	123.28

Intramolecular Geometry

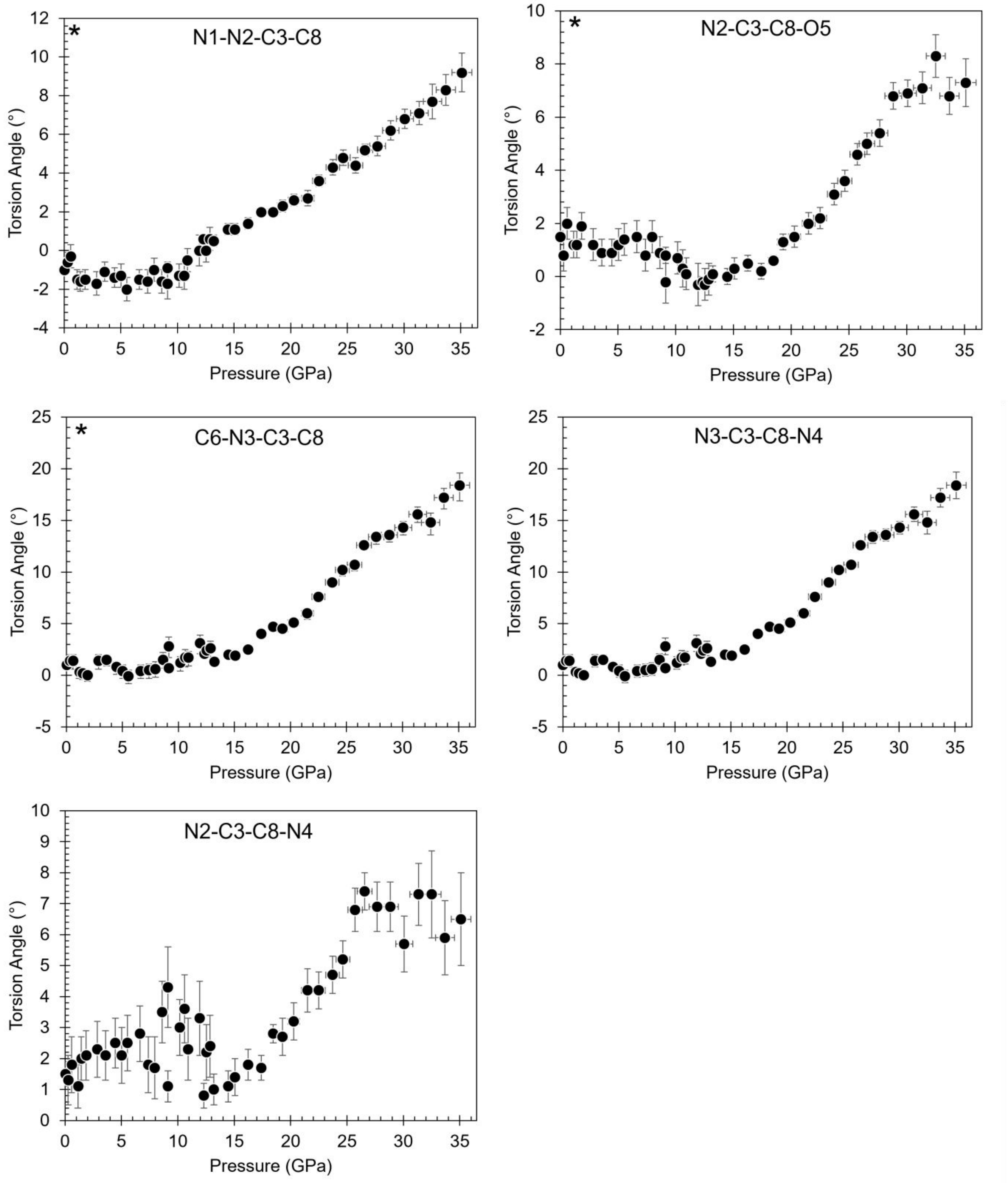

Figure S5 *Selected torsion angles as a function of pressure. Asterisk indicates 'normalized' torsion angles where they wrap around 0 or 180°*

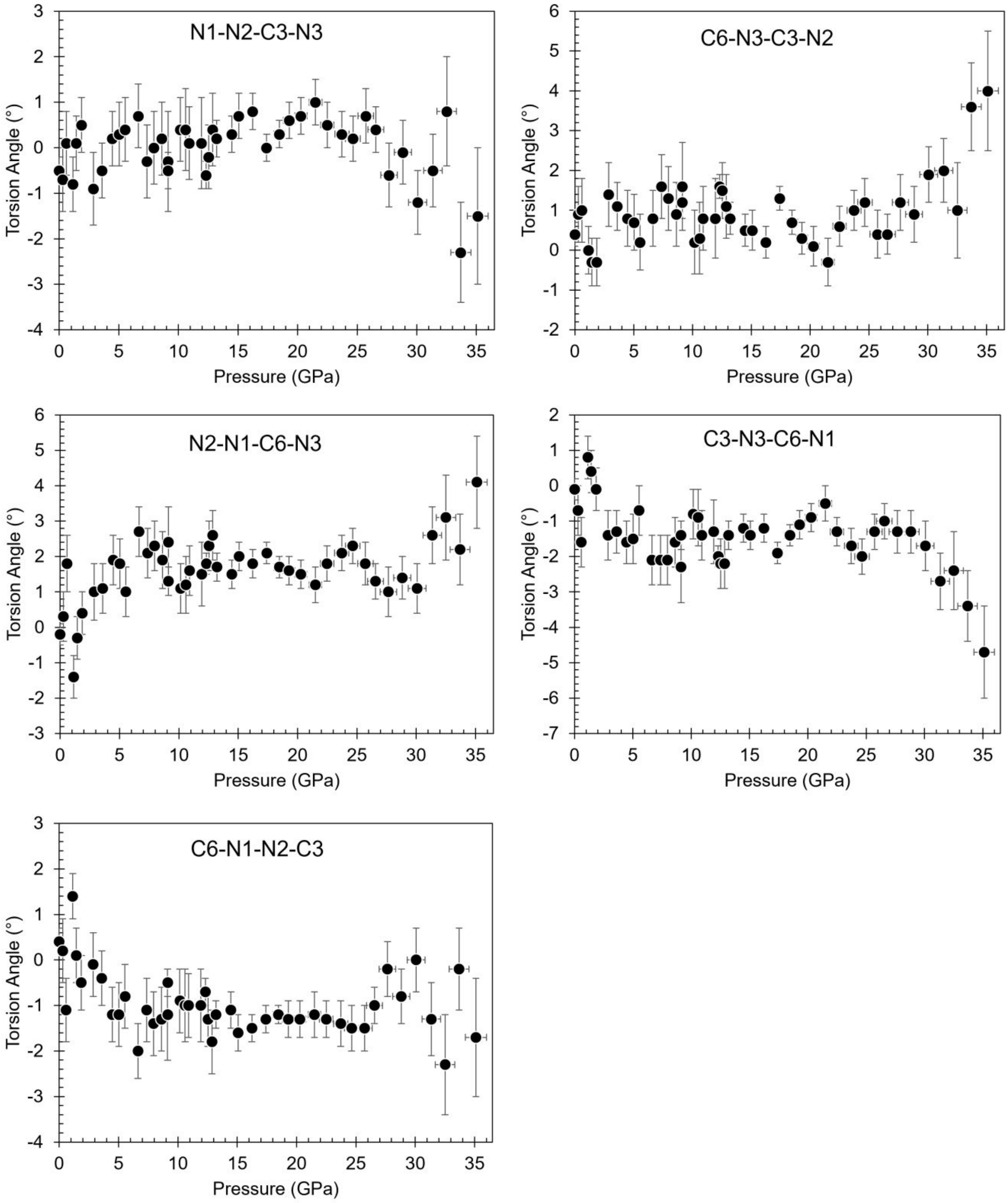

Figure S5 Continued *Selected torsion angles as a function of pressure. Asterisk indicates 'normalized' torsion angles where they wrap around 0 or 180°.*

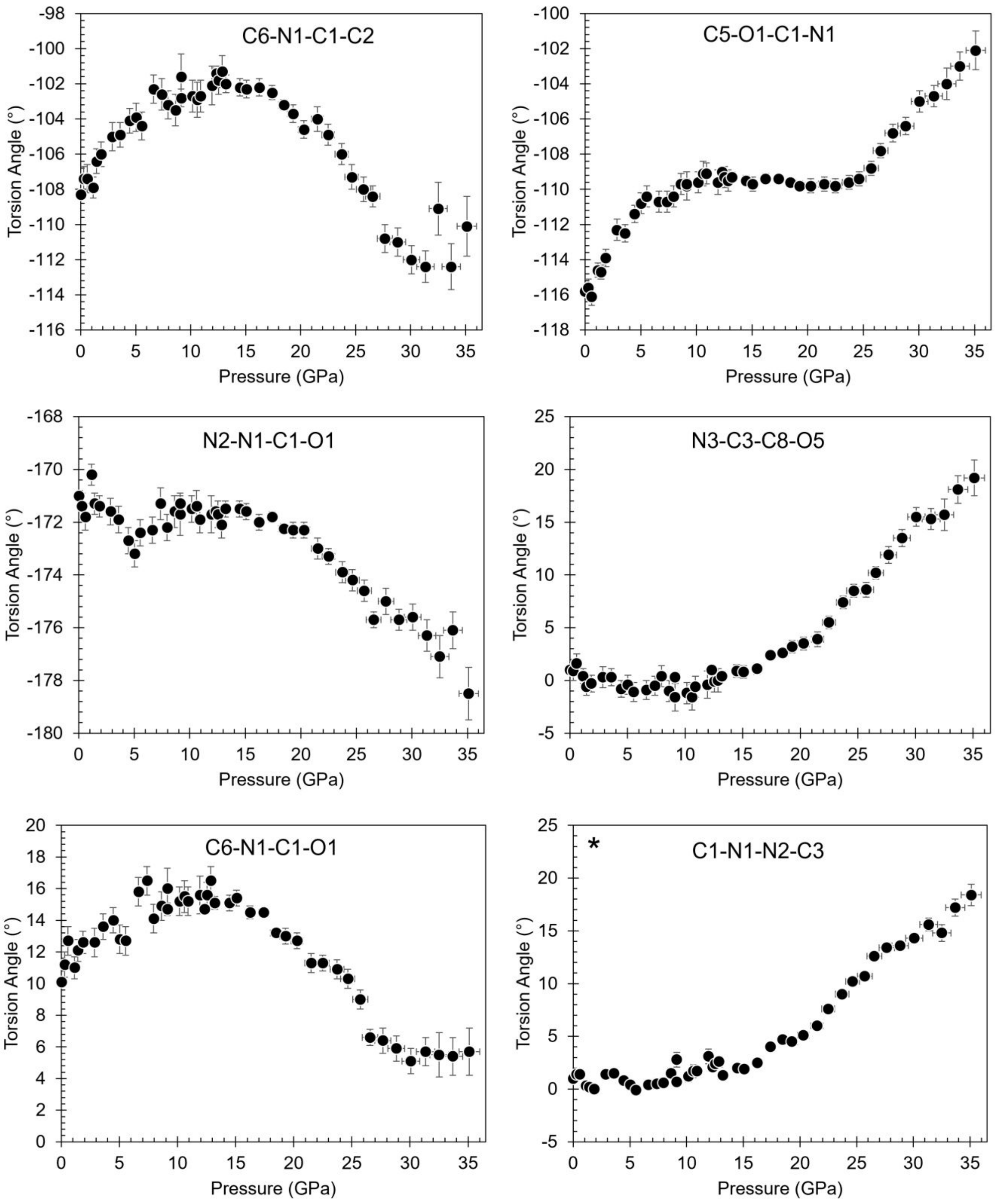

Figure S5 Continued *Selected torsion angles as a function of pressure. Asterisk indicates 'normalized' torsion angles where they wrap around 0 or 180°.*

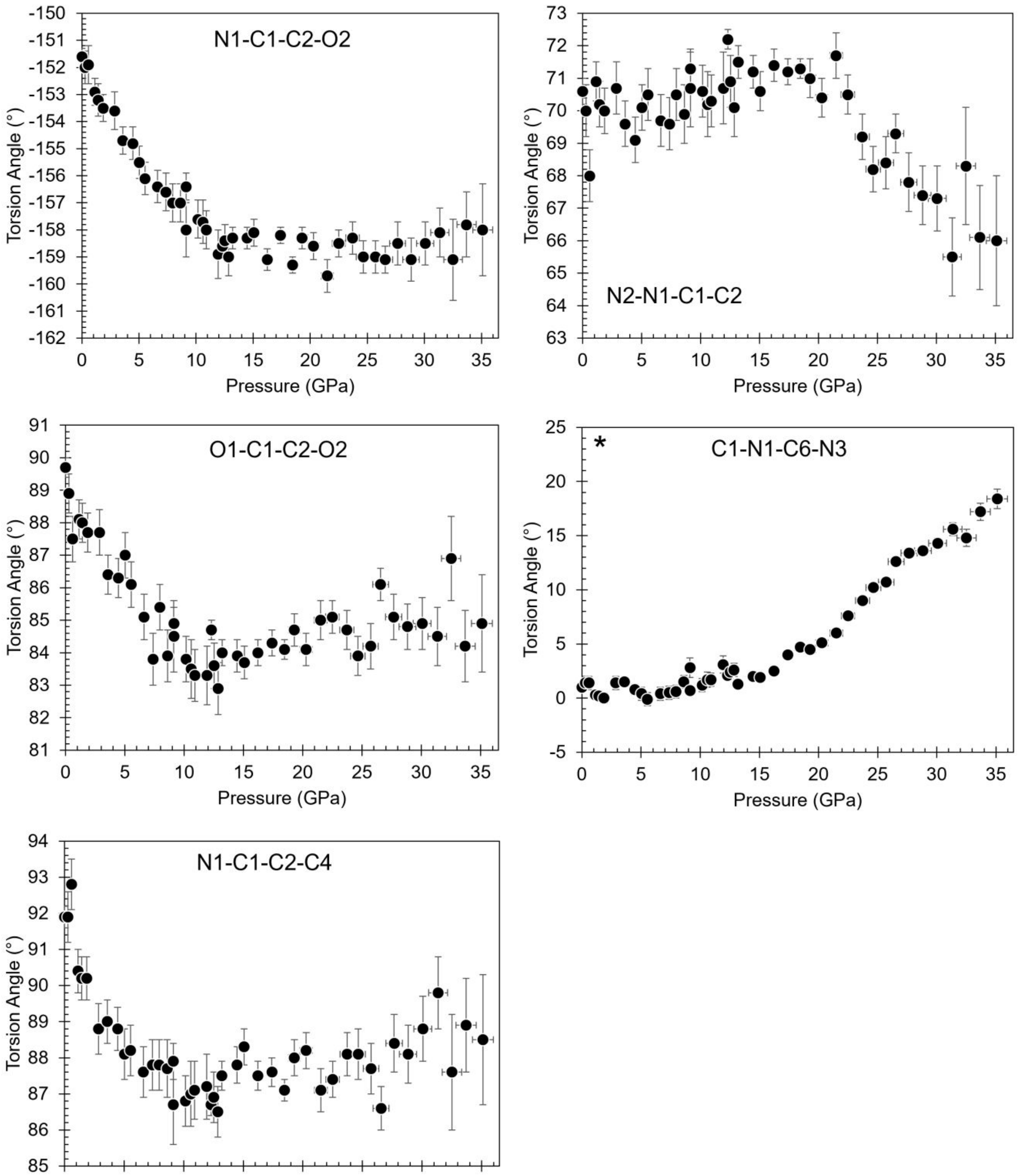

Figure S5 Continued *Selected torsion angles as a function of pressure. Asterisk indicates 'normalized' torsion angles where they wrap around 0 or 180°.*

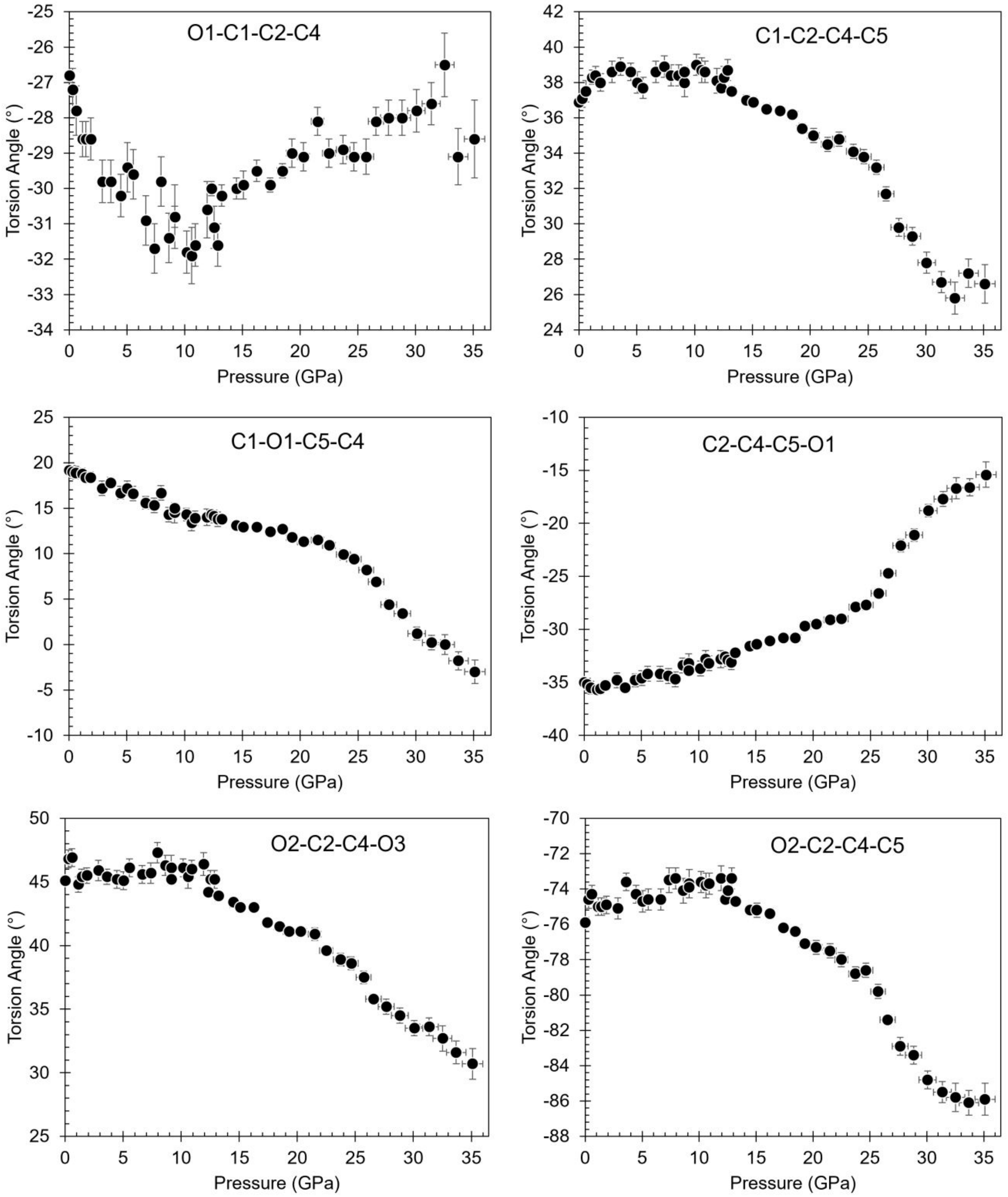

Figure S5 Continued *Selected torsion angles as a function of pressure. Asterisk indicates 'normalized' torsion angles where they wrap around 0 or 180°.*

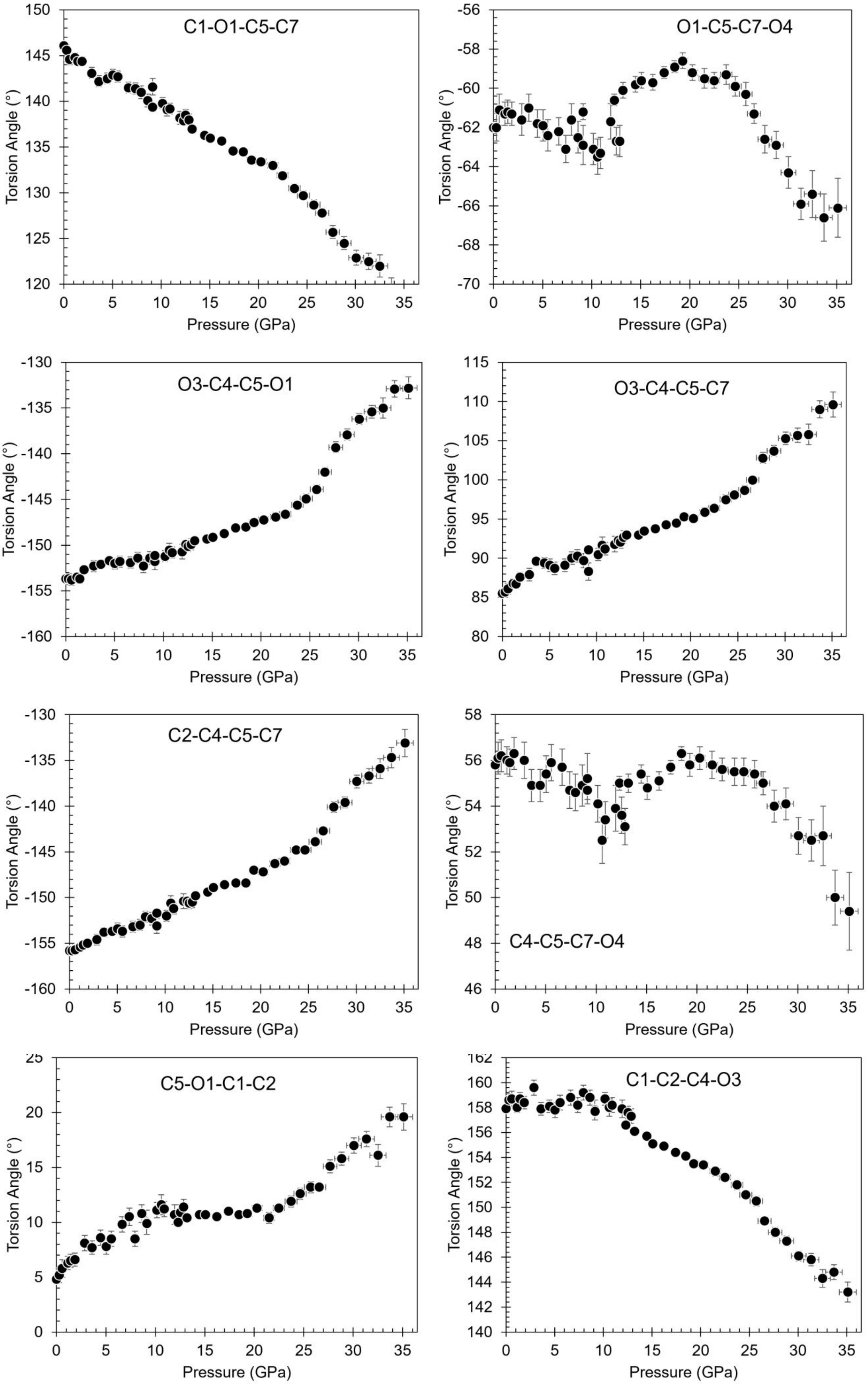

Figure S5 Continued *Selected torsion angles as a function of pressure. Asterisk indicates 'normalized' torsion angles where they wrap around 0 or 180°.*

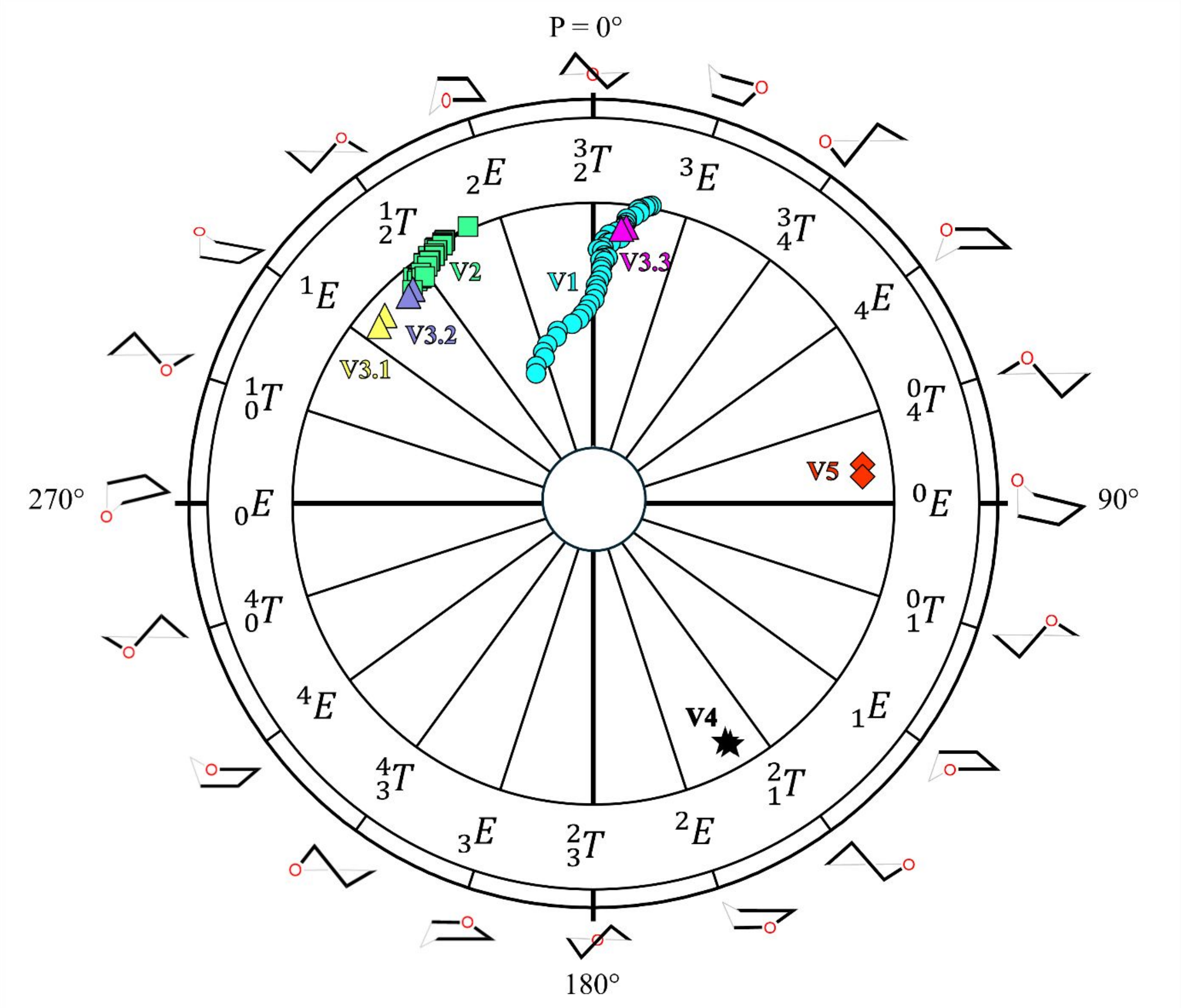

Figure S6 *Pseudorotation wheel for the ribofuranosyl sugar pucker, showing the idealized envelope (E) and twist (T) conformations and their associated pseudorotation phase angles, P. Experimental pseudorotation phase angles are shown for ribavirin* ***V1*** *from this work and for the* ***V2–V5*** *phases reported previously in ref. 15. Labels* ***V3.1–V3.3*** *denote the three crystallographically independent molecules in the* ***V3*** *phase (Z' = 3). Angular position corresponds to P, while radial position is scaled linearly with pressure using the* ***V1*** *compression range, 0–35.1 GPa, as reference; pressure increases inward. The* ***V1*** *compression series follows a continuous trajectory following the $E_3 \rightarrow T^3_2 \rightarrow E_2$ pathway. Conformation labels follow the standard furanose pseudorotation convention.*

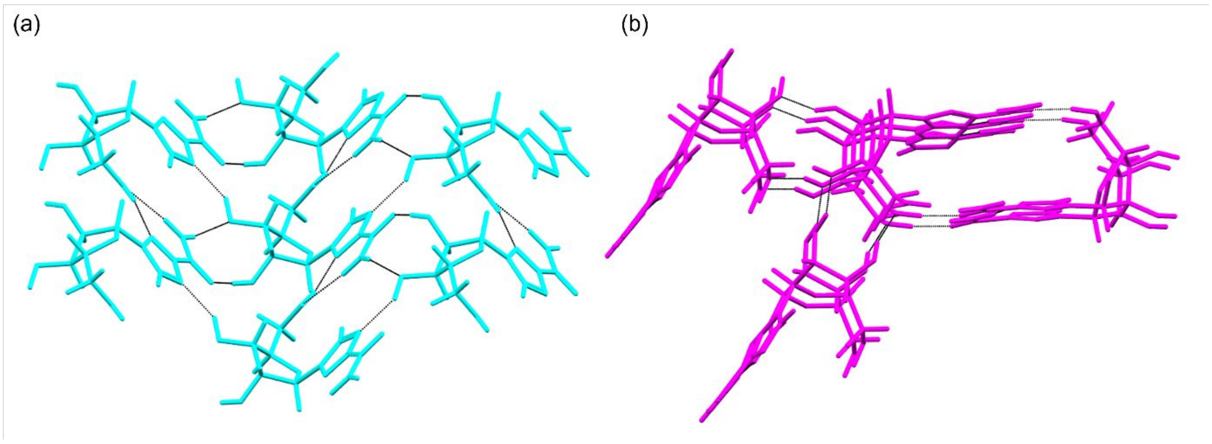

Figure S7 *Comparison of the ambient-pressure hydrogen-bonded packing motifs in ribavirin (a)* ***V1*** *and (b)* ***V2****. Molecules are shown in single colors for clarity and hydrogen bonds are shown as black dashed lines. The two polymorphs adopt distinct supramolecular arrangements, providing different packing constraints on the ribofuranosyl group during compression.*

Intermolecular Interaction Energies PIXEL and SAPT0

Table S4 *Comparison of total intermolecular interaction energies (kJ mol $^{-1}$) calculated using PIXEL and SAPT0 for contacts A/A'–G/G' at 0 and 35.1 GPa. Values are averaged over symmetry-equivalent contacts. |ΔE| represents the absolute difference between PIXEL and SAPT0 energies. Deviations increase at high pressure, particularly for strongly compressed contacts, reflecting methodological differences in the treatment of short-range repulsion.*

Interaction	0 GPa			35.1 GPa		
	PIXEL	SAPT0	|ΔE|	PIXEL	SAPT0	|ΔE|
A/A'	−51.2	−57.3	6.1	2.1	−34.4	36.5
B/B'	−47.6	−48.7	1.1	19.1	−10.0	29.1
C/C'	−40.1	−31.2	8.9	50.8	34.0	16.8
D/D'	−35.9	−33.5	2.4	73.9	29.8	44.1
E/E'	−30.6	−30.3	0.3	93.7	18.7	75.0
F/F'	−16.3	−14.1	2.2	27.2	0.7	26.5
G/G'	−12.8	−10.9	1.9	22.5	4.3	18.2

A/A': (−x + 3/2, −y + 1, z − 1/2) and (−x + 3/2, −y + 1, z + 1/2); B/B': (−x + 1/2, −y + 1, z − 1/2) and (−x + 1/2, −y + 1, z + 1/2); C/C': (x − 1, y, z) and (x + 1, y, z); D/D': (−x + 1, y − 1/2, −z + 3/2) and (−x + 1, y + 1/2, −z + 3/2); E/E': (x − 1/2, −y + 1/2, −z + 1) and (x + 1/2, −y + 1/2, −z + 1); F/F': (x + 1/2, −y + 3/2, −z + 1) and (x − 1/2, −y + 3/2, −z + 1); G/G': (−x, y − 1/2, −z + 3/2) and (−x, y + 1/2, −z + 3/2).

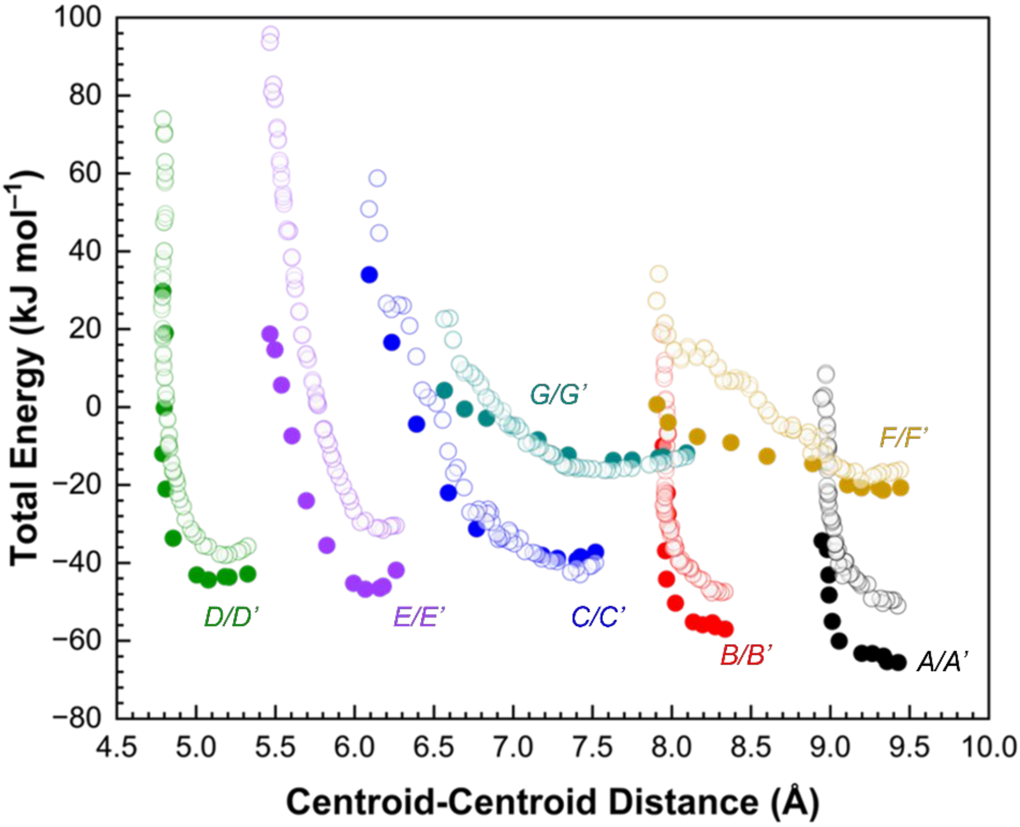

Figure S8 *Comparison of total intermolecular interaction energies calculated using SAPT0 (solid circles) and PIXEL (open circles) for contacts A/A'–G/G' as a function of centroid–centroid separation. Contact labels correspond to those listed in* ***Table S4****. Although SAPT0 yields smaller absolute energies, both methods reproduce the same ordering and distance-dependent trends.*

Table S5 *Total PIXEL interaction energies (kJ mol⁻¹) averaged over symmetry-equivalent interactions (A/A'–G/G') as a function of pressure.*

Pressure (GPa)	*A/A'*	*B/B'*	*C/C'*	*D/D'*	*E/E'*	*F/F'*	*G/G'*
0.0	-51.2	-47.6	-40.1	-35.9	-30.6	-16.3	-12.8
0.3	-49.8	-47.8	-40.9	-36.8	-30.6	-16.6	-13.2
0.6	-49.8	-47.4	-41.1	-37.3	-30.8	-17.1	-13.5
1.1	-49.6	-47.0	-43.1	-38.0	-31.7	-16.8	-14.3
1.4	-50.5	-47.8	-41.5	-38.2	-31.2	-17.4	-14.6
1.9	-49.3	-46.9	-42.5	-37.9	-31.2	-17.9	-14.9
2.9	-49.3	-44.6	-39.8	-36.0	-29.3	-19.0	-15.8
3.6	-45.7	-44.3	-39.6	-35.8	-29.7	-16.7	-15.8
4.5	-45.8	-43.1	-39.1	-33.3	-26.8	-16.7	-16.3
5.0	-45.0	-41.8	-37.8	-32.6	-24.3	-16.3	-16.3
5.5	-44.4	-41.4	-37.5	-31.5	-23.1	-15.8	-16.3
6.6	-42.8	-40.8	-37.2	-29.0	-20.5	-14.8	-16.1
7.4	-43.1	-41.0	-33.9	-25.7	-18.1	-15.5	-15.9
8.0	-39.9	-39.3	-35.2	-23.9	-16.3	-12.2	-15.8
8.6	-41.3	-39.5	-32.5	-22.2	-13.5	-11.5	-15.9
9.1	-39.8	-39.9	-34.6	-19.8	-12.5	-12.0	-15.4
9.1	-43.4	-36.3	-31.8	-18.5	-9.8	-7.7	-15.4
10.2	-40.6	-36.4	-33.6	-16.9	-8.5	-8.8	-15.7
10.6	-36.9	-34.9	-34.1	-16.4	-5.7	-6.6	-15.2
10.9	-37.5	-36.4	-32.6	-14.4	-5.9	-7.8	-14.9
11.9	-37.3	-30.9	-29.7	-10.7	0.4	-6.0	-12.8
12.3	-34.8	-31.2	-27.2	-7.3	3.5	-5.6	-12.7
12.5	-35.4	-32.5	-26.4	-9.5	0.8	-6.0	-12.2
12.9	-35.3	-32.0	-27.5	-10.0	1.4	-4.9	-12.2
13.2	-31.3	-30.5	-29.6	-3.3	6.5	-4.8	-11.9
14.5	-29.4	-27.1	-26.4	1.8	12.2	-2.3	-10.5
15.1	-29.5	-27.7	-27.4	3.4	13.5	-1.0	-10.7
16.2	-28.5	-27.4	-27.1	7.5	18.4	1.7	-9.2
17.4	-25.7	-25.9	-20.8	10.5	24.4	5.1	-9.5
18.5	-24.3	-25.4	-15.7	13.4	30.4	6.6	-6.2
19.3	-22.2	-22.9	-17.1	17.7	33.1	7.0	-5.0
20.3	-22.5	-21.1	-11.5	19.2	38.3	6.6	-5.1
21.5	-21.1	-22.4	-3.4	25.5	45.2	6.8	-4.5
22.5	-15.8	-15.9	0.8	28.2	45.3	10.0	-2.0
23.7	-14.2	-13.4	2.4	33.3	52.6	12.3	-0.4
24.6	-14.2	-10.1	4.2	37.5	54.3	15.0	1.3
25.7	-10.9	-6.9	12.9	40.0	59.2	12.9	2.5
26.6	-6.1	-6.8	20.8	47.4	62.8	15.1	5.6
27.7	-10.4	-7.2	25.9	49.0	68.6	12.3	7.5
28.8	-10.1	-6.8	26.2	57.9	71.6	14.5	8.6
30.1	-4.7	-1.4	25.0	60.1	79.1	18.3	9.1
31.4	-0.7	1.7	26.5	62.9	82.8	21.5	11.0
32.5	2.7	7.7	44.6	70.6	80.9	19.0	17.2
33.7	8.4	11.3	58.7	70.1	95.6	34.1	22.7
35.1	2.1	19.1	50.8	73.9	93.7	27.2	22.5

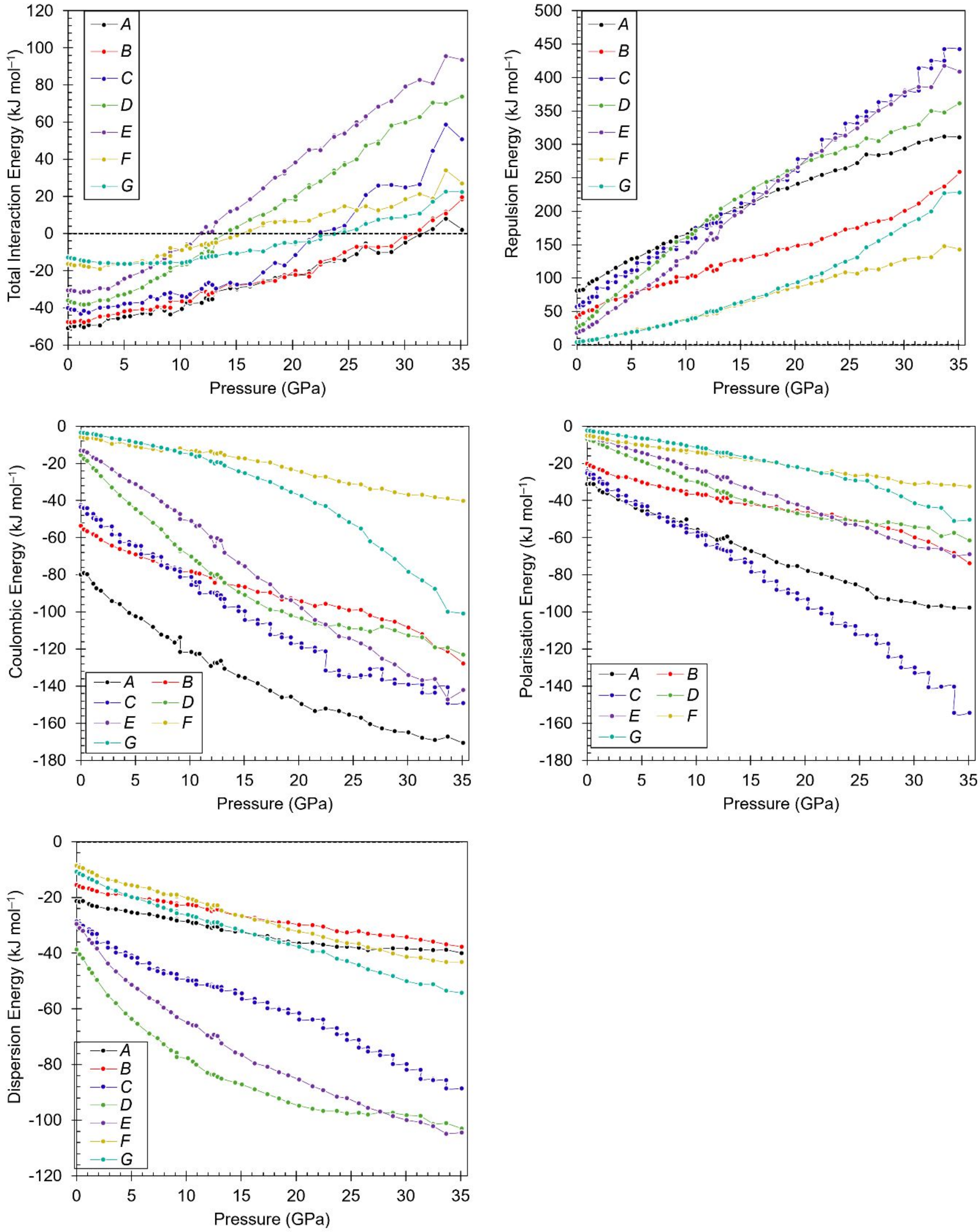

***Figure S9** Pressure dependence of PIXEL-derived interaction energy components for contacts A/A'–G/G'. Panels show (top left) total interaction energy, (top right) exchange-repulsion, (middle left) Coulombic, (middle right) polarization, and (bottom) dispersion contributions as a function of pressure. Increasing pressure produces monotonic growth of the repulsive term for all contacts, which ultimately dominates the total interaction energy despite increasingly stabilizing Coulombic and dispersion contributions. Energies are reported as averages over symmetry-equivalent contacts.*

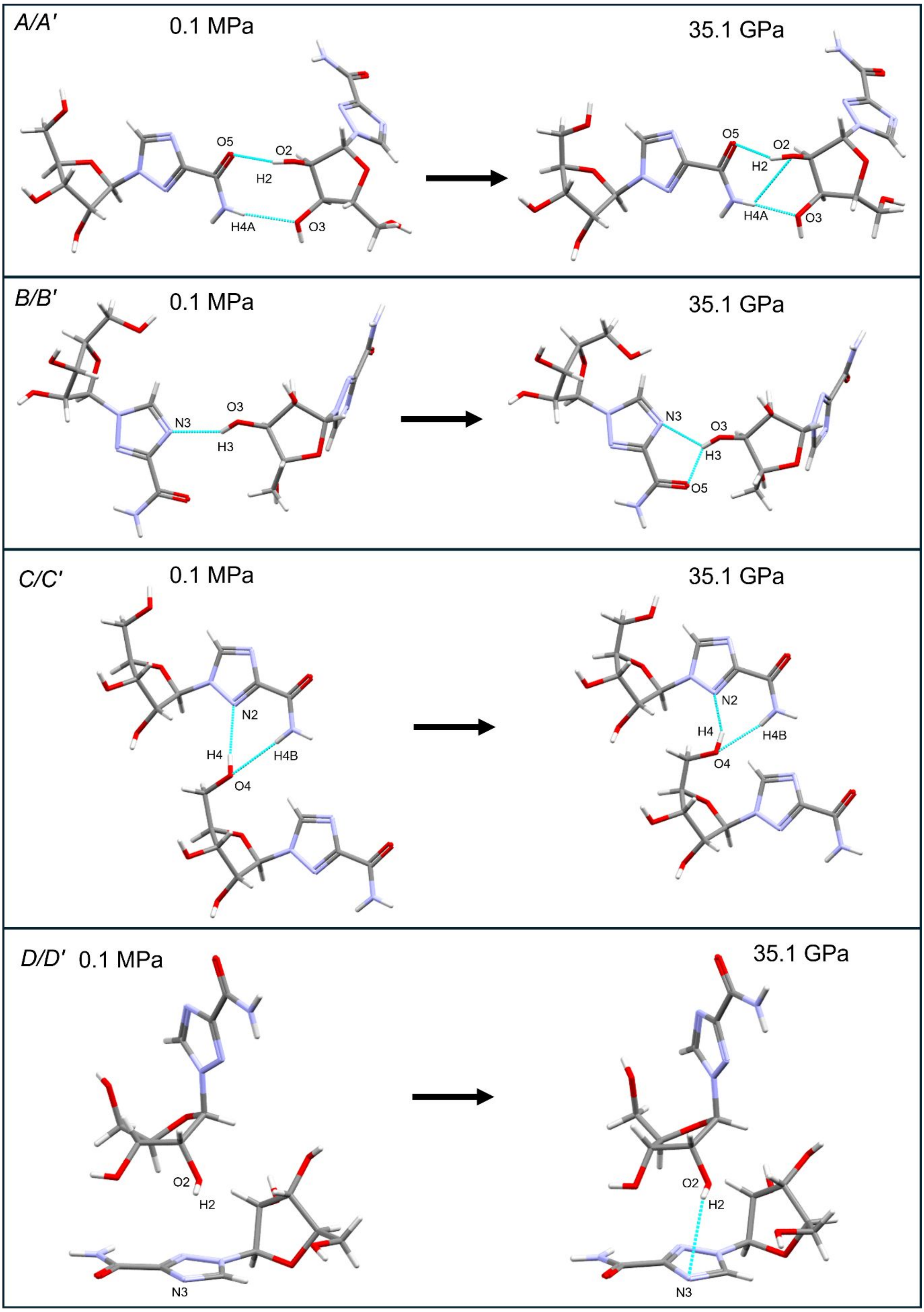

Figure S10 *Comparison of selected intermolecular interactions in ribavirin V1 at ambient pressure (0.1 MPa) and 35.1 GPa. Interactions A/A', B/B', C/C' and D/D' within the first coordination sphere are shown. Hydrogen-bond contacts are indicated by cyan dashed lines. Interaction B/B' becomes bifurcated at high pressure, while new contacts are observed for A/A' and D/D' at 35.1 GPa. Molecular geometries correspond to the experimentally determined crystal structures at each pressure.*

Table S6 *Donor–acceptor distances (D···A) and D–H···A angles for selected hydrogen-bond contacts at 0.1 MPa and 35.1 GPa. An en dash (–) indicates that the contact is not present at the corresponding pressure. Values marked with an asterisk fall outside the typical statistical range observed for comparable donor–acceptor motifs in the CSD. The corresponding interaction motifs are shown in* ***Fig. S8****.*

		**D···A (Å)**		**Angle (°)**	
**Interaction**	**Donor → Acceptor**	**0.1 MPa**	**35.1 GPa**	**0.1 MPa**	**35.1 GPa**
A/A'	N4 → O3	2.99	2.61*	151.9	160.8
	O2 → O5	2.69	2.41*	162.0	151.7
	N4 → O2	–	3.13	–	122.3*
B/B'	O3 → N3	2.88	2.55*	145.3	123.0*
	O3 → O5	–	2.41*	–	141.2*
C/C'	N4 → O4	3.26	2.98	174.4	158.5
	O4 → N2	2.92	2.48*	161.4	152.2*
D/D'	O2 → N3	–	3.23*	–	121.9*

References

(1) Macrae, C. F.; Sovago, I.; Cottrell, S. J.; Galek, P. T. A.; McCabe, P.; Pidcock, E.; Platings, M.; Shields, G. P.; Stevens, J. S.; Towler, M.; Wood, P. A. Mercury 4.0: From Visualization to Analysis, Design and Prediction. *J. Appl. Crystallogr.* **2020**, *53* (1), 226–235. https://doi.org/10.1107/S1600576719014092.

(2) Agati, M.; Romi, S.; Fanetti, S.; Radacki, K.; Hanfland, M.; Braunschweig, H.; Marder, T. B.; Clark, S. J.; Friedrich, A.; Bini, R. Insights into Topochemical versus Stress-Induced High-Pressure Reactivity of Azobenzene by Single Crystal X-Ray Diffraction. *Chem. Sci.* **2025**, *16* (21), 9240–9254. https://doi.org/10.1039/D5SC00432B.

(3) Zhou, W.; Aslandukov, A.; Minchenkova, A.; Hanfland, M.; Dubrovinsky, L.; Dubrovinskaia, N. Structural Transformations and Stability of Benzo[a]Pyrene under High Pressure. *IUCrJ* **2025**, *12* (1), 16–22. https://doi.org/10.1107/S2052252524010455.

(4) Montisci, F.; Ernst, M.; Macchi, P. Experimental and Computational Study on the Effects of High Pressure on the Crystal Structure of Boron Nitrilotriacetate. *Cryst. Growth Des.* **2023**, *23* (4), 2745–2754. https://doi.org/10.1021/acs.cgd.2c01532.

(5) Friedrich, A.; Collings, I. E.; Dziubek, K. F.; Fanetti, S.; Radacki, K.; Ruiz-Fuertes, J.; Pellicer-Porres, J.; Hanfland, M.; Sieh, D.; Bini, R.; Clark, S. J.; Marder, T. B. Pressure-Induced Polymerization of Polycyclic Arene–Perfluoroarene Cocrystals: Single Crystal X-Ray Diffraction Studies, Reaction Kinetics, and Design of Columnar Hydrofluorocarbons. *J. Am. Chem. Soc.* **2020**, *142* (44), 18907–18923. https://doi.org/10.1021/jacs.0c09021.

(6) Collings, I. E.; Hanfland, M. Packing Rearrangements in 4-Hydroxycyanobenzene Under Pressure. *Molecules* **2019**, *24* (9), 1759. https://doi.org/10.3390/molecules24091759.

(7) Eikeland, E.; Thomsen, M. K.; Overgaard, J.; Spackman, M. A.; Iversen, B. B. Intermolecular Interaction Energies in Hydroquinone Clathrates at High Pressure. *Cryst. Growth Des.* **2017**, *17* (7), 3834–3846. https://doi.org/10.1021/acs.cgd.7b00408.

(8) Giordano, N.; Beavers, C. M.; Kamenev, K. V.; Marshall, W. G.; Moggach, S. A.; Patterson, S. D.; Teat, S. J.; Warren, J. E.; Wood, P. A.; Parsons, S. High-Pressure Polymorphism in L-Threonine between Ambient Pressure and 22 GPa. *CrystEngComm* **2019**, *21* (30), 4444–4456. https://doi.org/10.1039/C9CE00388F.

(9) Guńka, P. A.; Olejniczak, A.; Fanetti, S.; Bini, R.; Collings, I. E.; Svitlyk, V.; Dziubek, K. F. Crystal Structure and Non-Hydrostatic Stress-Induced Phase Transition of Urotropine Under High Pressure. *Chem. – Eur. J.* **2021**, *27* (3), 1094–1102. https://doi.org/10.1002/chem.202003928.

(10) Laniel, D.; Fedotenko, T.; Winkler, B.; Aslandukova, A.; Aslandukov, A.; Aprilis, G.; Chariton, S.; Milman, V.; Prakapenka, V.; Dubrovinsky, L.; Dubrovinskaia, N. A Reentrant Phase Transition and a Novel Polymorph Revealed in High-Pressure Investigations of CF4 up to 46.5 GPa. *J. Chem. Phys.* **2022**, *156* (4), 044503. https://doi.org/10.1063/5.0079402.

(11) Nakagawa, T.; Zhang, C.; Bu, K.; Dalladay-Simpson, P.; Vrankić, M.; Bolton, S.; Laniel, D.; Wang, D.; Liang, A.; Ishii, H.; Hiraoka, N.; Garbarino, G.; Rosa, A. D.; Hu, Q.; Lü, X.; Mao, H.; Ding, Y. Narrowing Band Gap Chemically and Physically: Conductive Dense Hydrocarbon. *Commun. Mater.* **2025**, *6* (1), 98. https://doi.org/10.1038/s43246-025-00814-2.

(12) Zhou, W.; Yin, Y.; Laniel, D.; Aslandukov, A.; Bykova, E.; Pakhomova, A.; Hanfland, M.; Poreba, T.; Mezouar, M.; Dubrovinsky, L.; Dubrovinskaia, N. Polymorphism of Pyrene on Compression to 35 GPa in a Diamond Anvil Cell. *Commun. Chem.* **2024**, *7* (1), 209. https://doi.org/10.1038/s42004-024-01294-0.

(13) Zhou, W.; Li, X.; Akbar, F. I.; Pakhomova, A.; Hanfland, M.; Dubrovinsky, L.; Dubrovinskaia, N. Compressional Behavior of Naphthalene (C10H8) and Anthracene (C14H10) up to 50 GPa. *ACS Omega* **2025**, *10* (42), 50230–50242. https://doi.org/10.1021/acsomega.5c06935.

(14) Agati, M.; Romi, S.; Fanetti, S.; Garbarino, G.; Haines, J.; Bini, R. High-Pressure Structural and Electronic Properties of Bibenzyl (1,2-Diphenylethane) from Synchrotron SC-XRD and Two-Photon-Induced Fluorescence. *Cryst. Growth Des.* **2026**, *26* (2), 970–984. https://doi.org/10.1021/acs.cgd.5c01569.

(15) Tiwari, B.; Liermann, H.-P.; Parsons, S.; Giordano, N. High-Pressure Polymorphism of Ribavirin. *Cryst. Growth Des.* **2025**, *25* (10), 3537–3547. https://doi.org/10.1021/acs.cgd.5c00372.
